# Transport Oligonucleotides—A Novel System for Intracellular Delivery of Antisense Therapeutics

**DOI:** 10.3390/molecules25163663

**Published:** 2020-08-11

**Authors:** Oleg V. Markov, Anton V. Filatov, Maxim S. Kupryushkin, Ivan V. Chernikov, Olga A. Patutina, Anton A. Strunov, Elena L. Chernolovskaya, Valentin V. Vlassov, Dmitrii V. Pyshnyi, Marina A. Zenkova

**Affiliations:** 1 Institute of Chemical Biology and Fundamental Medicine SB RAS, Lavrentieva ave. 8, 630090 Novosibirsk, Russia; markov_ov@niboch.nsc.ru (O.V.M.); antonfeelatoff@gmail.com (A.V.F.); kuprummax@gmail.com (M.S.K.); chernikovivanv@gmail.com (I.V.C.); patutina@niboch.nsc.ru (O.A.P.); elena_ch@niboch.nsc.ru (E.L.C.); valentin.vlassov@niboch.nsc.ru (V.V.V.); pyshnyi@niboch.nsc.ru (D.V.P.); 2 Institute of Cytology and Genetics SB RAS, Lavrentieva ave. 10, 630090 Novosibirsk, Russia; strunov.anton@gmail.com

**Keywords:** antisense oligonucleotide, lipophilic oligonucleotide conjugates, delivery, transport oligonucleotide, multiple drug resistance

## Abstract

Biological activity of antisense oligonucleotides (asON), especially those with a neutral backbone, is often attenuated by poor cellular accumulation. In the present proof-of-concept study, we propose a novel delivery system for asONs which implies the delivery of modified antisense oligonucleotides by so-called transport oligonucleotides (tON), which are oligodeoxyribonucleotides complementary to asON conjugated with hydrophobic dodecyl moieties. Two types of tONs, bearing at the 5′-end up to three dodecyl residues attached through non-nucleotide inserts (TD series) or anchored directly to internucleotidic phosphate (TP series), were synthesized. tONs with three dodecyl residues efficiently delivered asON to cells without any signs of cytotoxicity and provided a transfection efficacy comparable to that achieved using Lipofectamine 2000. We found that, in the case of tON with three dodecyl residues, some tON/asON duplexes were excreted from the cells within extracellular vesicles at late stages of transfection. We confirmed the high efficacy of the novel and demonstrated that *MDR1* mRNA targeted asON delivered by tON with three dodecyl residues significantly reduced the level of P-glycoprotein and increased the sensitivity of KB-8-5 human carcinoma cells to vinblastine. The obtained results demonstrate the efficacy of lipophilic oligonucleotide carriers and shows they are potentially capable of intracellular delivery of any kind of antisense oligonucleotides.

## 1. Introduction

Nucleic acid-based therapy is highly promising approach to treat a variety of diseases. The high specificity of therapeutic nucleic acids (antisense oligonucleotides, siRNA, antagomirs, etc.) in silencing particular targets, along with their limited number of side effects, places them at the forefront of drug development, with several examples already in clinical trials [1,2] and approved for therapeutic use [3,4,5,6]. However, the challenges associated with their use, including the delivery of therapeutic nucleic acids into target cell/tissues and the short duration of their silencing effects, attributed to insufficient nuclease resistance, currently limit the application of these therapeutics [7]. Numerous systems for oligonucleotide delivery have been proposed, including viral vectors and non-viral delivery agents, such as liposomes [8,9] polymeric [10,11], and metal nanoparticles [12,13]. Some of these systems allow efficient delivery of oligonucleotides into cultured cells, however, they have significant side effects when applied in vivo. For instance, viral vectors are immunogenic and can induce mutagenesis; liposomes and polyplexes containing cationic lipids or polymers often exhibit cytotoxicity or/and stimulate immune system; and metal nanoparticles are accumulated in cells and tissues and are not metabolized.

The conjugation of nucleic acids with various ligands stimulating their accumulation in the target cells is one of the most promising approaches for their delivery. Conjugates of oligonucleotides represent the latest products, such as IONIS’s LICA (ligand-conjugated antisense) platform [14,15] as well as Alnylam’s most recently commercialized siRNA-based drug Givlaari [16], and all drugs in the pipeline [17] created on the GalNAc (*N*-acetylgalactosamine) platform. *N*-acetylgalactosamine specifically delivers nucleic acids to the liver with high efficiency [1], therefore, the problem of delivery of nucleic acids to the liver may be considered to be resolved. However, specific delivery of nucleic acids to other organs is complicated by the necessity to find specific receptors present on the cell surface in these organs. The other promising strategy for solving this problem is to conjugate nucleic acids with lipophilic molecules, since these interact in the bloodstream with lipoproteins whose receptors are expressed throughout the body [18]. Moreover, the binding of lipophilic conjugates with lipoproteins reduces their excretion by the kidneys and increases their circulation time in the bloodstream [19,20].

While circulating in the bloodstream, nucleic acids remain sensitive to nucleases. Since phosphate is directly involved in nucleic acid cleavage, there were attempts to introduce chemical modifications to this position, such as phosphorothioate (PS) [21], boranophosphate [22], N3 phosphoramidate [23], dimethylethylenediamine [24], phosphonoacetate [25], *tert*-butyl-S-acyl-2-thioethyl [26], phosphorylguanidine [27], and mesyl [28], as well as analogues of nucleic acids with modified structures of the furanose cycle, such as morpholino [29] and peptide nucleic acid [30], increasing the nuclease resistance of the preparations. However, today the most frequently used phosphate stabilizing modification of antisense oligonucleotides (asON) is PS, which can cause toxicity due to non-specific interactions of PS-modified oligonucleotides with cell proteins [31,32,33]. Recent attempts to reduce non-specific binding of PS oligonucleotides to cellular proteins and improve the therapeutic index of fully PS modified asON by controlling phosphorothioate chirality have not been very successful [34]. Substitution of PS in asON to create a charge-neutral modification, such as methylphosphonate or methoxypropylphosphonate linkages, can reduce or eliminate toxicity of PS-modified asON [35]. Among the novel phosphate modifications, those obtained by the Staudinger reaction, phosphoryl guanidines and mesyl, look to be the most promising [27,28]. Therefore, in this work, we used a new, non-toxic, charge-neutral phosphoryl guanidine modification (PX) to stabilize the phosphate of the asON [27].

To improve cellular uptake, asONs are conjugated with various delivery-promoting molecules, including cholesterol [36], polyethylene glycol [37,38], α-tocopherol [39,40], cell-penetrating peptides [41,42], etc. Despite the perspective of this approach, the redundant modifications of asONs with such molecules, especially in combination with modifications of the oligonucleotide backbone to improve their nuclease resistance, can significantly affect their biological activity, create problems in adaptation of length and composition of linker groups and, finally, complicate the scheme of asON synthesis and isolation as well as increase their cost. On the other hand, it was demonstrated that formation by asONs various supramolecular structures, such as concatemeric complexes with partially complementary lipophilic oligonucleotides [43,44] or aggregates [45], significantly enhances their intracellular accumulation and bio-performance.

In the present proof-of-concept study, we propose a novel simple and inexpensive approach for intracellular delivery of asON, gene-directed oligonucleotides or oligonucleotides with any other function, based on the use of transport oligonucleotides (tON). tONs are oligonucleotides complementary to the oligonucleotide to be delivered (here and after, dON) and conjugated with dodecyl residues. Such delivery systems allow the different functional modifications on two molecules to be split; dON can contain only modifications providing nuclease resistance, whereas transport oligonucleotide (tON) contains moieties promoting intracellular delivery. The hydrophobicity of the carrier can be modulated by changing the number of hydrophobic of groups. We found that tONs with two and three dodecyl residues efficiently delivered dONs, including those with a neutral sugar-phosphate backbone, to cells without being cytotoxic. We confirmed a high efficacy of the novel transport oligonucleotide-based delivery system using *MDR1* mRNA as a target and demonstrated that *MDR1* mRNA targeted asON delivered by tON with three dodecyl residues significantly reduced the level of P-glycoprotein and increased the sensitivity of KB-8-5 human carcinoma cells to vinblastine.

## 2. Results

### 2.1. Design and Synthesis of Transport Oligonucleotides

It is well documented that attachment of various hydrophobic molecules (cholesterol, for example) to antisense oligonucleotides significantly improves the uptake of such oligonucleotide conjugates by cells, however, this can affect its bioperformance and intracellular localization (see reviews [46,47]). In this work, we designed a novel delivery system for antisense oligonucleotides lacking the disadvantages of asON conjugates. This system consists of antisense oligonucleotides bearing only those modifications required for its nuclease resistance and bioperformance and transport oligonucleotides equipped with hydrophobic moieties whose only function is to deliver the complementary antisense oligonucleotide into the cells.

To test this concept we used a simple hydrophobic moiety, a dodecyl residue, and synthesized two series of transport oligonucleotides (hereinafter, tON), bearing from one to three dodecyl residues, in order to investigate the influence of stepwise increases in hydrophobicity of tON on cellular uptake of tON alone as well as duplexes formed by tON and complementary delivered oligonucleotides (dON). The choice of a dodecyl residue as a hydrophobic group is based on its “moderate” hydrophobicity, which allows the regulation of the properties of tON by changing the position and number of the aliphatic moieties within the oligonucleotide chain.

**Figure 1:**
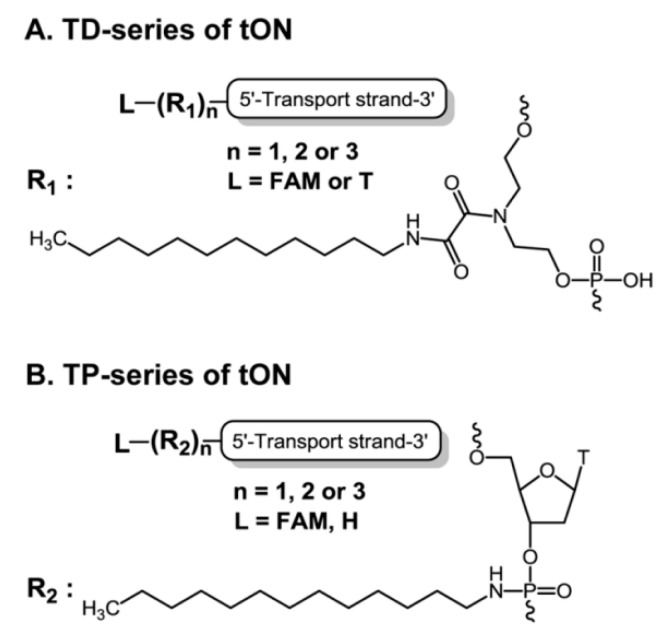
Structures of transport oligonucleotides of (**A**) TD- and (**B**) TP-series.

**Figure 2:**
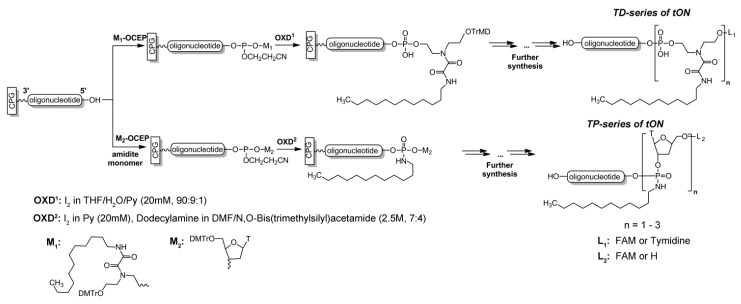
Scheme of synthesis of transport oligonucleotides.

Two convenient approaches which can be applied without significant changes in the procedure of oligonucleotide solid phase synthesis were used to introduce dodecyl residues into an arbitrary position on the oligonucleotide chain. In the first approach, a custom non-nucleosidic phosphoramidite monomer, obtained according to a recently published protocol [48], was utilized. With the use of the monomer bearing a dodecylamine residue as a side chain, tONs containing up to three non-nucleosidic units can be obtained with reasonable yield (up to 50%). The conventional phosphoramidite synthons allow one to introduce the hydrophobic moieties in the desired positions of the oligonucleotide chain, as well as additionally decorate the derivatives with reporter groups (Figure 1 and Figure 2).

For the second approach, a set of tONs were obtained based on a directed Arbuzov-type reaction method [49], leading to the formation of phosphoramidate linkages under the oxidative coupling of an amine with a phosphite triester, which is the conventional intermediate obtained after a condensation procedure in automated oligonucleotide synthesis. Slight changes in the synthetic protocol, i.e., replacement of typical aqueous iodine to an anhydrous dodecylamine/I_2_ mixture at the oxidation step of the just-formed phosphite internucleotide linkage in the lengthened oligonucleotide chain, yielded functionalized phosphoramidate at the desired position. This synthetic trick can be easily repeated several times during the oligonucleotide synthesis and modified oligonucleotides bearing up to three contiguously introduced dodecylamine units can be obtained with an acceptable yield (Figure 1 and Figure 2). Both approaches permit combination of dodecylamine insertion with any additional derivatizations which are commonly used in solid support synthesis of oligonucleotides, including labeling with fluorophores. Depending on the synthetic scheme used, synthesized tONs were designated as TD1–TD3 or TP1–TP3 for schemes 1 and 2, correspondingly (Figure 2). Sequences, modification, and designations of oligonucleotides used in this work are listed in Table 1. Importantly, a tON tethering three non-nucleosidic units possesses a high level of hydrophobicity which is comparable with the hydrophobicity of oligonucleotides equipped with a cholesterol residue [48]. This is one of the reasons why more than three dodecylamine residues are undesired. Another benefit of dodecyl-containing derivatives is the significant increase in retention time upon HPLC isolation (Supplementary Material, Figure S1).

A set of oligonucleotides containing from one to three dodecyl residues with 5′-terminal localization of non-nucleotide units was synthesized. As a fluorescent label, fluorescein (FAM) or cyanine dye (By5.5) residues were introduced into the tON structure using a commercially available 5′-modifier. To avoid the undesirable presence of a free hydroxyethyl moiety as a part of the non-nucleotide backbone (degradation under alkaline conditions, [48]), in the case of synthesis of unlabeled oligonucleotides an additional thymidine monomer was introduced at the 5′-terminal position (Figure 2).

A 17-mer oligonucleotide, having no molecular targets in the human and rodent genome, but forming a stable complementary duplex with the corresponding tON, was selected as the dON. The scrambled sequence of the dON was chosen to avoid any effects on cells other than those of tONs. To compare the effectiveness of intracellular accumulation of dON with different backbones, three types of dONs were synthetized: phosphodiester oligonucleotide (PO), the well-studied analogue phosphorotioate (PS), and a new type of oligonucleotide analogue with an electrically neutral backbone, phosphoryl guanidine oligonucleotide (PX). Thus, the ability of tONs to deliver both negatively-charged (PO and PS) and neutral (PX) oligonucleotides could be evaluated.

The melting temperatures (T_m_) of the duplexes formed by dON (PO series) and tONs (TD1–TD3 and TP1–TP3) under physiological conditions showed negligible changes in the thermal stability of complementary complexes, regardless of the amount of introduced dodecyl residues. The T_m_ of the duplex formed by the parent transport oligonucleotide devoid of hydrophobic residues with FAM-ON-PO is 60 °C and only a slight increase in T_m_ is observed for duplexes FAM-ON-PO/TD1–TD3. On average, terminal hydrophobization of the duplexes resulted in an approximately +1 °C increase in T_m_ per dodecyl unit. Thus, hydrophobic modification has only a minimal effect on the affinity of the oligonucleotides to the complementary sequence. Additionally, it should be noted that both types of tONs, i.e., those with non-nucleosidic insert (TD-series) and with phosphoramidate internucleotide linkage (TP-series), are potentially biodegradable. One can be cleaved under mild acidic conditions and the other by phosphodiesterase action.

### 2.2. Cell Viability Assay

**Figure 3:**
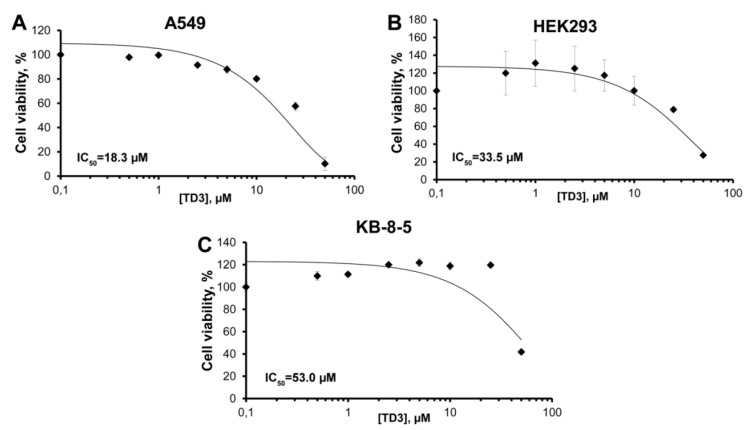
Dose-response curves of TD3 for A549 (**A**), HEK293 (**B**) and KB-8-5 (**C**) cells. Cells were incubated with increasing concentrations of TD3 for 24 h. Cell viability was measured by an xCELLigence instrument in real-time mode. The results are expressed as a percentage of viable cells observed after treatment with TD3 vs. control cells (100%) incubated in the absence of TD3. All experimental points were run in triplicate for statistical analysis. Data are presented as MEAN ± SD.

Transport oligonucleotides with hydrophobic groups could be expected to interact with cell membranes and affect cell functions. To study tON cytotoxicity, as the most hydrophobic member, the TD3 oligonucleotide was selected (the highest retention time in HPLC). The in vitro cytotoxicity of TD3 with respect to A549, HEK293 and KB-8-5 cells was studied in real-time mode by using an xCELLigence instrument (ACEA Biosciences, Santa Clara, CA, USA) for 24 h (Supplementary Material, Figure S2). Based on the data on cell viability, the dose-response curves for TD3 were built (Figure 3) and IC_50_ values were evaluated. It was seen that TD3, at high concentrations, could affect the viability of the cells, with IC_50_ values showing low cytotoxicity (53.0 and 33.5 µM) for KB-8-5 and HEK293 cells, respectively, and moderate cytotoxicity (IC_50_ 18.3 µM) for A549 cells. The multidrug resistant KB-8-5 cells were less sensitive to TD3 than other drug-sensitive cells (Figure 3). It is worth mentioning that the IC_50_ values for all three cell lines under the study were significantly higher than the concentration of asONs usually used in cell culture experiments, which is 1–5 µM. Thus, in the range of concentration required for asON delivery, which is below 5 µM, tON (TD3) is non-toxic for the cells.

### 2.3. Intracellular Accumulation of tON in A549 and HEK293 Cells

To assess the ability of tONs to enter the cells in the absence of any delivery vehicles, the intracellular accumulation of FAM-labeled tONs in A549, KB-8-5 and HEK293 cells was studied by flow cytometry. Human embryonic kidney HEK293 cells were used because these cells have been widely used in experiments with various transfectants [43,50,51,52,53,54,55]. Human epidermoid carcinoma cells KB-8-5 exhibiting multi-drug resistance phenotype were used because several types of antisense oligonucleotides and siRNAs were targeted to *MDR1* mRNA overexpressed by these cells [56,57,58,59,60]. Human lung carcinoma A549 cells were chosen as possible target cells of tumor origin.

**Figure 4:**
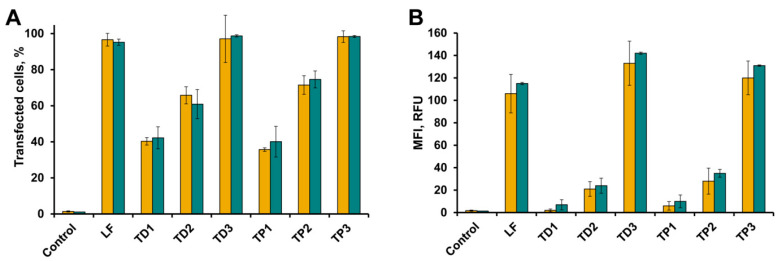
Intracellular accumulation of FAM-labeled transport oligonucleotides into A549 (yellow bars) and HEK293 (green bars) cells. The percentage of fluorescent cells (**A**) and mean fluorescence intensity (**B**) were measured using flow cytometry 4 h post transfection. LF = transfection of FAM-ON-PO mediated by Lipofectamine 2000. Data presented as MEAN ± SD.

Cells were incubated in DMEM in the presence of FAM-tON for 4 h followed by flow cytometry analysis (Figure 4A,B). As shown in Figure 4, the transfection efficiency (TE) (number of FAM positive cells in population) and mean fluorescence intensities (MFI) of cells showing intracellular accumulation of tON was directly correlated with the number of hydrophobic dodecyl residues in their structure: an increase in the number of dodecyl residues from one to three regardless of the type of attachment (R1 or R2) resulted in a significant increase in intracellular accumulation of the respective oligonucleotide. Transport oligonucleotides TD3 and TP3 very efficiently accumulated both in HEK293 and A549 cells—up to 100% of cells were transfected with an MFI of 130 RFU. It should be emphasized that TD3 and TP3 had TE comparable to that achieved using Lipofectamine 2000 (LF), considered as the “gold standard” of transfection (Figure 4A,B). TD2 and TP2 were less efficient, especially in terms of the amount of oligonucleotide accumulated in the cells (MFI) (70–80% of transfected cells with 20–40 RFU), while TD1 and TP1 with one dodecyl residue exhibited the lowest transfection efficiency. Similar levels of intracelluler accumulation of tONs were observed for KB-8-5 cells in terms of both number of transfected cells and fluorescence intensity (data not shown).

### 2.4. Accumulation of tON/dON Duplexes in A549 and HEK293 Cells

Since TD3 and TP3 very efficiently penetrated into the cells, we used these tONs to study how duplex formation between model dON and tON could stimulate penetration of dON into the cells. As mentioned above, dON-model antisense oligonucleotide had a random sequence and was specially constructed not to affect gene expression in the transfected cells. dONs with different sugar phosphate backbone modifications were used in this series of experiments: ON-PO, ON-PS, and ON-PX (Table 1). The TE of tON/dON duplexes with respect to HEK293, A549 cells and KB-8-5 was evaluated using FAM-labeled dONs.

**Figure 5:**
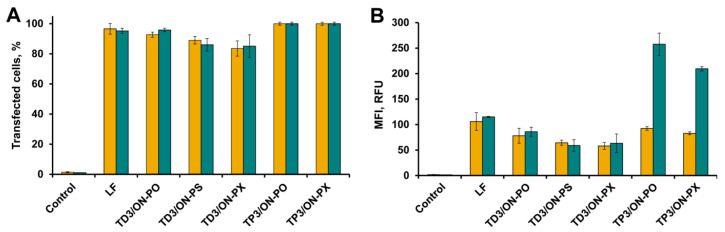
Transport oligonucleotide mediated accumulation of FAM-dONs bearing different sugar phosphate backbone modifications in A549 (yellow bars) and HEK293 (green bars) cells. The percentage of fluorescent cells (**A**) and mean fluorescence intensity (**B**) were measured using flow cytometry 4 h post transfection. LF = transfection of FAM-ON-PO mediated by Lipofectamine 2000. Data presented as the mean ± SD.

We found (Figure 5A,B) that FAM-dONs were efficiently delivered into the cells in the duplexes with TD3 regardless of the type of their backbone modification, including those with a charge-neutral phosphoryl guanidine. TD3 delivered FAM-dONs with an efficiency comparable to that of Lipofectamine 2000 (LF), and 90–100% of cells in population were transfected. However, although the mean fluorescence intensity (MFI) of the cells transfected with the duplexes was high (from 50 to 80 RFU), nevertheless this was 1.3–2 times lower in comparison with Lipofectamine-mediated delivery of FAM-ON-PO.

TP3 delivered FAM-dONs with approximately the same efficiency as TD3 in A549 (100% of cells, 90–100 RFU) and KB-8-5 cells (data not shown). In the case of HEK293 cells, an abnormally high mean fluorescence intensity of FAM-dONs delivered by TP3 was observed (Figure 5B) that exceeded the level of LF-mediated delivery of FAM-ON-PO by 2–2.5 fold. The abnormal TE of duplexes TP3/dON observed for HEK293, but not for A549 and KB-8-5 cells, does not correlate with MFI levels of FAM-TP3 intracellular accumulation (Figure 4B) and the reasons for this fact are not clear. These results, together with the highly reproducible efficiency of transfection mediated by TD type of tON, forced us to exclude TP the type of tON from further research and subsequent experiments were performed with TD type of tON (TD2 and TD3).

It should be noted that modifications of the sugar-phosphate backbone of dON did not influence the level of intracellular accumulation of tON/dON duplexes: TEs as well as MFIs of all three duplexes were almost equal (Figure 5). Since oligonucleotides containing phosphorothioate modifications could interact with intracellular proteins and phosphorothioate oligonucleotides are toxic to cells at concentrations over 5 × 10^−6^ M [32], only non-modified ON-PO and phosphoryl guanidine-modified ON-PX were selected for further experiments.

KB-8-5 cells were selected for further investigations because as it was mentioned above a number of antisense oligonucleotides and siRNAs were targeted *MDR1* mRNA overexpressed by these cells [56,57,58,59,60]. Thus these cells represent a well-studied model to determine the silencing activity of antisense oligonucleotides delivered by tONs.

### 2.5. Intracellular Localization of tON/dON Duplexes

**Figure 6:**
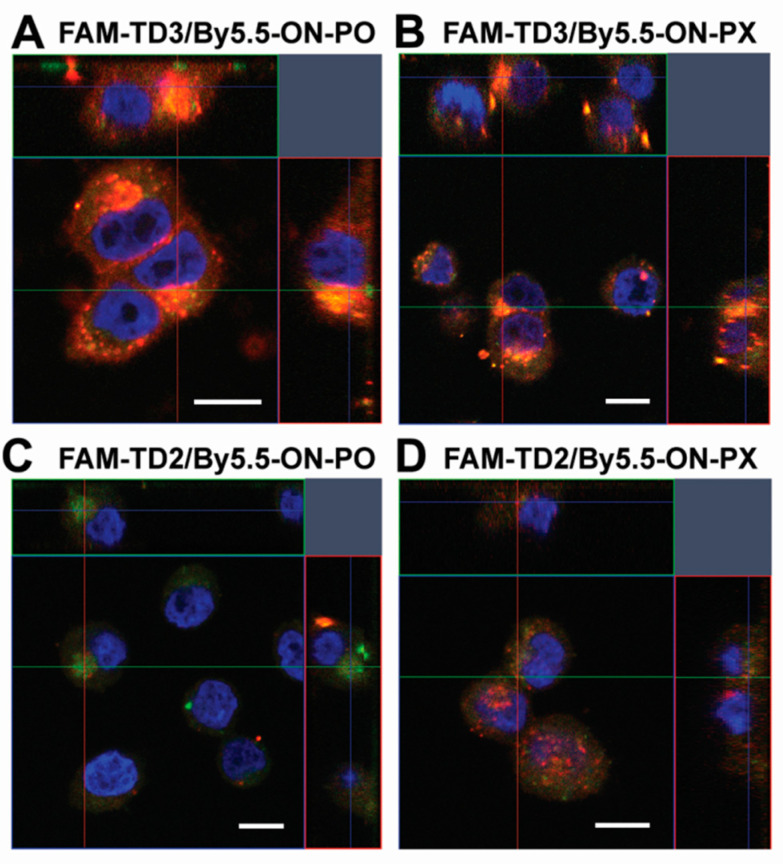
Intracellular localization of duplexes of transport oligonucleotide/antisense oligonucleotide in KB-8-5 cells. (**A)** FAM-TD3/By5.5-ON-PO. (**B**) FAM-TD3/By5.5-ON-PX. (**C**) FAM-TD2/By5.5-ON-PO. (**D**) FAM-TD2/By5.5-ON-PX. Analysis was performed by confocal fluorescent microscopy (Plan-apochromat 63×/1.40 Oil DIC M27 objective) 4 h post transfection. Data are presented as orthogonal projections. Blue signal = fluorescence of DAPI (nuclei staining), green signal = fluorescence of FAM-tONs, red signal = fluorescence of By5.5-dONs. Scale bar: 10 µm.

To find out whether the tON/asON duplexes were bound on cell membrane or accumulated in the cytoplasm we performed a confocal fluorescent microscopic study of the localization of duplexes tON/asON in KB-8-5 cells which will be further used to evaluate the biological activity of this delivery system (Figure 6). In these experiments tONs labeled with FAM (FAM-TD3 and FAM-TD2) and dON labeled with By5.5 (By5.5-ON-PO and By5.5-ON-PX) were used, and confocal microscopic analysis was performed 4 h post transfection.

Orthogonal projections of Z-stack images of KB-8-5 cells transfected with duplexes are presented in Figure 6. The orange signal that is combined signal from the green channel corresponding to FAM-labeled tONs and red channel corresponding to By5.5-labeled dONs is located in the cytoplasm of the cells in large granules and is absent or reduced in the nuclei (Figure 6). It should be mentioned that complexes of nucleic acids with Lipofectamine 2000 are also located in the cell cytoplasm in the form of similar granules [61]. From the presented data, it can be concluded that tON is fully co-localized with dON inside the cells. It is worth mentioning that TD3 delivered dON into KB-8-5 cells more efficiently as compared to TD2, regardless of the type of sugar phosphate backbone modifications of dON, which correlated with the flow cytometry data (Figure 5A,B).

### 2.6. Kinetics of tON/dON Duplex Accumulation in KB-8-5 Cells

#### 2.6.1. Flow Cytometry

Kinetics of accumulation of tON/dON duplexes in KB-8-5 cells was investigated by flow cytometry and confocal microscopy. Analysis of transfected cells was performed 1, 2, 4, 8, 16, and 24 h after addition of the duplex to the cells. To visualize the oligonucleotides under study, tONs were labeled with FAM (FAM-TD3 and FAM-TD2) and dONs were labeled with By5.5 (By5.5-ON-PO and By5.5-ON-PX). It worth mentioning that duplexes tON/dON were pre-formed prior to addition to the cells and analysis of duplex formation revealed that no free oligonucleotides (neither tON nor dON) were present in the mixture (data not shown).

**Figure 7:**
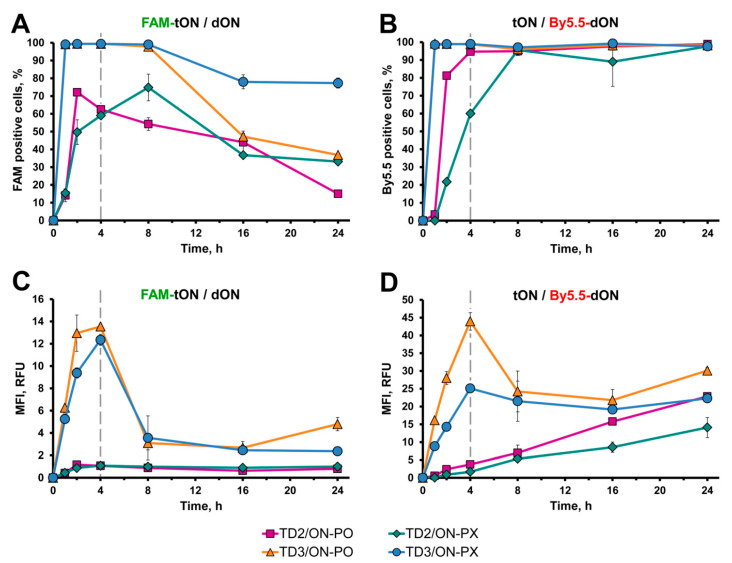
Kinetics of duplex accumulation into KB-8-5 cells. Analysis of samples was performed 1, 2, 4, 8, 16 and 24 h after addition of duplexes to the cells. Duplexes consisting of FAM-tON (TD3 or TD2) and By5.5-dON (ON-PO or ON-PX) were added to the cells at a final concentration of 1 µM. (**A**) The percentage of FAM-tON-positive cells. (**B**) The percentage of By5.5-dON-positive cells. (**C**) The mean fluorescence intensity of FAM-tON-positive cells. (**D**) The mean fluorescence intensity of By5.5-dON-positive cells. Duplexes TD2/ON-PO = magenta line with squares; TD2/ON-PX = green line with diamonds; TD3/ON-PO = orange line with triangles; TD3/ON-PX = blue line with circles. Dashed grey line = time point where FBS were added to the cells. Data presented as MEAN ± SD.

It could be seen that duplexes containing TD3 rapidly accumulated in 100% of cells: a plateau was reached within an hour after the addition of duplexes to the cells (Figure 7A,B orange and blue lines). In the course of further incubation, the intracellular accumulation of TD3 decreased to 80% for duplexes TD3/ON-PX and to 50–40% for TD3/ON-PO (Figure 7A, orange and blue lines). However, the same cell population was characterized by 100% of By5.5-positive cells, indicating that dONs remained in the cells during the 24 h of the experiment (Figure 7B, orange and blue lines). Duplexes containing TD2 were accumulated in cells less efficiently in comparison with TD3-containing ones; the plateau was reached in 4–8 h (Figure 7A,B, magenta and green lines) and the percentage of TD2-positive cells was 50–70% and gradually decreased to the level of 15–35% (Figure 7A,B, magenta and green lines). Similar to dON delivered in TD3/dON duplexes, the accumulation of dON mediated by TD2 reached the level of 100% of cells and remained at this level until 24 h (these kinetics were similar to that of TD3-containing duplexes) (Figure 7B, compare orange and blue with magenta and green lines).

The accumulation of duplexes (MFI values) revealed unusual kinetics. Duplexes containing TD3 efficiently accumulated in the cells during the first 4 h, both measured by FAM-TD3 or By5.5-dONs fluorescence; the mean florescence intensity reached 14 and 45 RFU, respectively (Figure 7C,D, orange and blue lines). Further incubation of cells resulted in a sharp decrease in TD3 accumulation (FAM fluorescence) and dON accumulation (By5.5 fluorescence). The intracellular content of TD3 reduced to the level of about 4 RFU, whereas a two-fold decrease in MFI corresponding to dONs was observed by 8 h. Then, these levels of MFI remained unchanged until the end of the experiment (Figure 7C,D, orange and blue lines).

Completely different kinetics of intracellular accumulation were observed for TD2-containing duplexes. The intensity of fluorescence of FAM-TD2 remained stably low (1 RFU) during the entire 24 h of incubation (Figure 7C, magenta and green lines), while intracellular accumulation of dONs mediated by TD2 gradually increased over 24 h, reaching MFI 10–20 RFU (Figure 7D, magenta and green lines). Interestingly, at the 24 h time point, the level of intracellular accumulation of both ON-PO and ON-PX mediated by TD2 was similar to that achieved in the presence of TD3, at 10–30 RFU (Figure 7D).

It should be mentioned that ON-PX-containing duplexes accumulated in cells less efficiently in comparison with ON-PO, both in the case of TD3 and TD2; approximately 15–40% differences during kinetics were observed from 0–24 h (Figure 7B,D compare orange with blue lines or magenta with green lines, respectively).

#### 2.6.2. Confocal Fluorescent Microscopy

**Figure 8:**
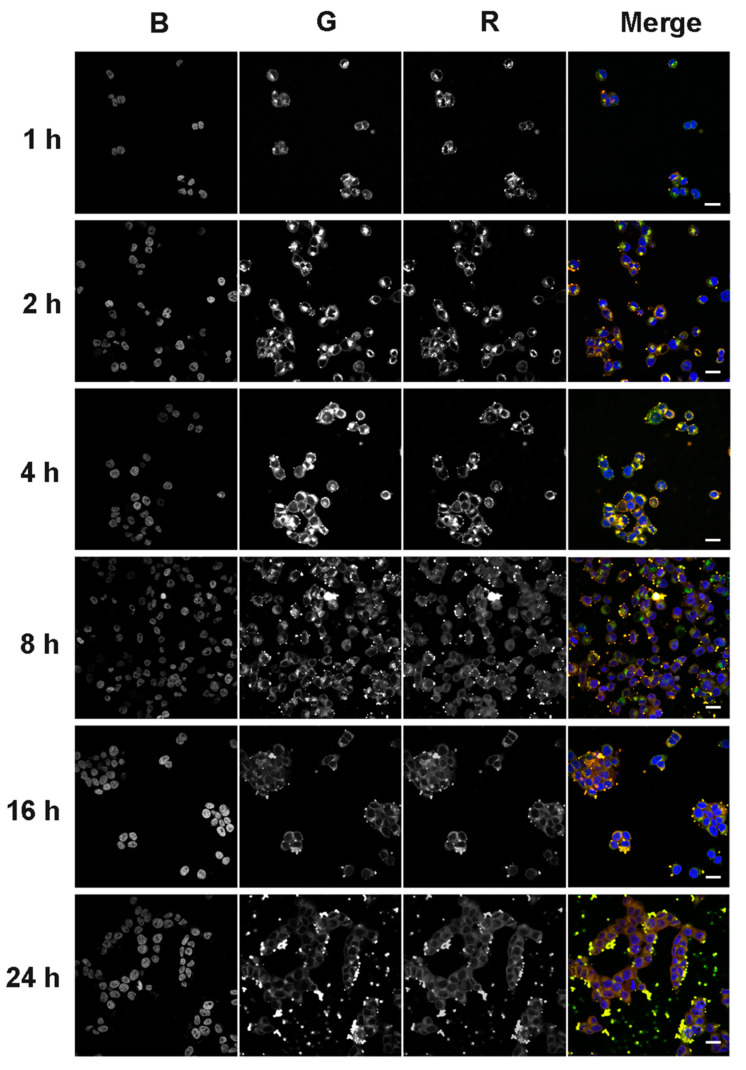
Accumulation of duplexes TD3/ON-PO in KB-8-5 cells. Analysis of samples was performed 1, 2, 4, 8, 16 and 24 h after addition of duplexes (1 µM) to the cells. Duplexes consisted of FAM-labeled TD3 and By5.5-labeled ON-PO. Analysis was performed with confocal fluorescent microscopy by using a plan-apochromat 63×/1.40 Oil DIC M27 objective. Three-channel (BGR) pictures were obtained using staining by DAPI (nuclei staining) (**B**); FAM (**G**), attached to TD3, and By5.5 (**R**), attached to ON-PO. Scale bars: 20 µm.

**Figure 9:**
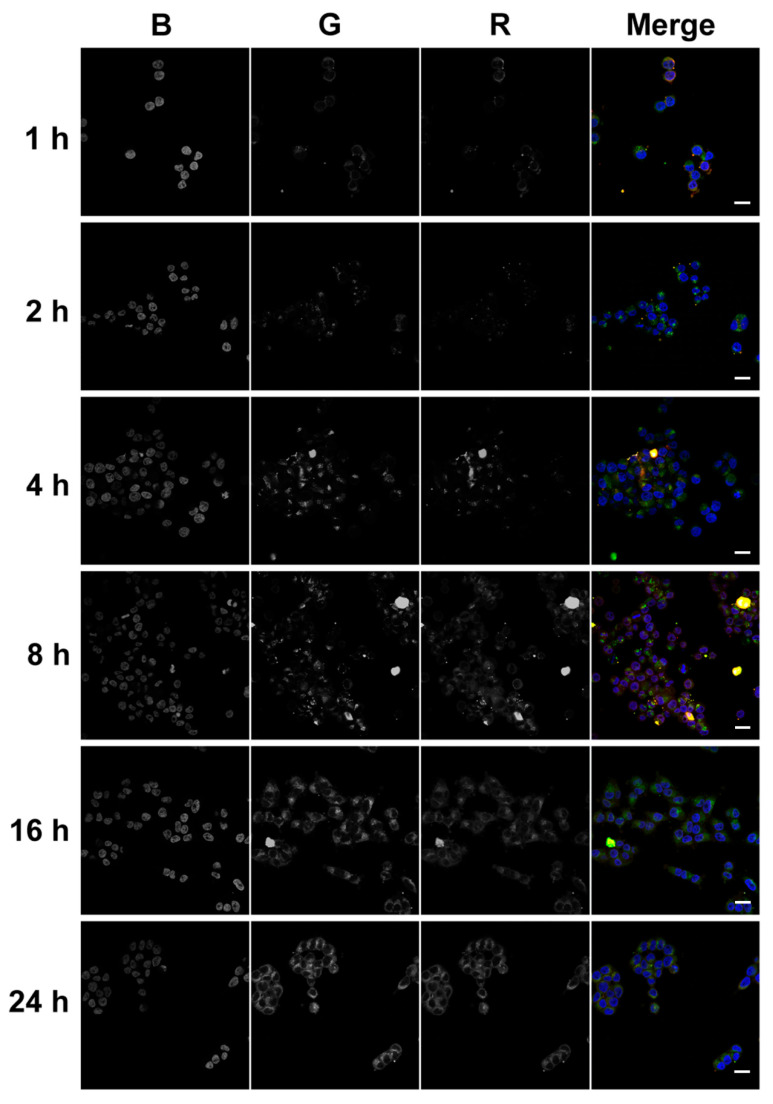
Accumulation of duplex TD2/ON-PO in KB-8-5 cells. Analysis of samples was performed 1, 2, 4, 8, 16 and 24 h after addition of duplexes (1 µM) to the cells. Duplexes consisted of FAM-labeled TD2 and By5.5-labeled ON-PO. Analysis was performed with confocal fluorescent microscopy by using a plan-apochromat 63×/1.40 Oil DIC M27 objective. Three-channel (BGR) pictures were obtained using staining by DAPI (nuclei staining) (**B**); FAM (**G**), attached to TD2, and By5.5 (**R**), attached to ON-PO. Scale bars: 20 µm.

Confocal microscopic analysis of the intracellular accumulation of tONs/dONs duplexes was performed at the same time points as flow cytometry analysis (1, 2, 4, 8, 16, and 24 h after duplex addition to KB-8-5 cells) with the same tONs (FAM-TD3 and FAM-TD2, green channel) and dONs (By5.5-ON-PO and By5.5-ON-PX, red channel). The typical kinetics of intracellular accumulation of TD3/ON-PO and TD2/ON-PO duplexes are presented in Figure 8 and Figure 9, respectively. Accumulation of corresponding ON-PX-containing duplexes was similar to that of ON-PO containing ones and is shown in Supplementary Information (Figures S3 and S4 for TD3/ON-PX and TD2/ON-PX, respectively).

It was seen that all tendencies of intracellular accumulation of duplexes observed by flow cytometry could also be seen by confocal microscopy. For instance, the maximal accumulation of TD3-containing duplexes was observed at the 4 h time point; afterwards the intensities of tON and dON signals decreased (Figure 8 and Figure S3). Kinetics of the intracellular accumulation of TD2-containing duplexes was characterized by the absence of any sharp decrease in the intensity of tON and dON signals (Figure 9 and Figure S4), however, total efficiency of its accumulation was significantly lower in comparison with TD3-containing duplexes (compare Figure 8 and Figure 9).

The data show that signals of tON and dON, in most cases, are co-localized, indicating that duplexes did not dissociate inside the cells. Apparently, this fact is related to the high melting temperature of duplexes. It should be mentioned that no fluorescence associated with the duplexes was found in the nuclei of cells during the entire 24 h of analysis, indicating their predominant cytoplasmic localization.

Surprisingly, starting from the 8 h time point, large amounts of TD3/dON-containing duplexes were observed in the extracellular space (Figure 8 and Figure S3), correlating with the decrease in fluorescence intensity of transfected cells according to flow cytometry data (Figure 7C,D).

### 2.7. On the Mechanism of Release of TD3/dON Duplexes from Cells

We can assume several mechanisms of formation of the observed granules in the extracellular space, these are: (1) the formation of aggregates of a hydrophobic duplex with serum proteins; (2) the induction of apoptosis duplexes and their incorporation into apoptotic bodies; or (3) the inclusion of duplexes in vesicles exocytozed from cells. Therefore, we tested these assumptions experimentally.

The ability of FBS components to bind duplexes was analyzed by confocal microscopy to check if TD3/dON duplexes located outside the cells at the late stages of transfection could be their aggregates with proteins of FBS. A total of 1 µM of FAM-TD3/By5.5-ON-PO duplexes were incubated on coverslips in 24-well plate with cultural medium supplemented with 10% FBS in the absence of cells for 16 h followed by confocal microscopic analysis. No aggregates of duplexes or their complexes with serum components were detected.

In order to exclude the accumulation of duplexes in apoptotic bodies, KB-8-5 cells incubated with 1 µM of TD3/ON-PO for 24 h were analyzed by an annexin-FITC apoptosis staining/detection kit (Abcam, Cambridge, UK). The data showed that incubation of cells with TD3/ON-PO duplexes for 24 h did not induce apoptosis of cells (Supplementary Material, Figure S6A). Furthermore, TD3/ON-PO duplexes did not affect the cell cycle (Supplementary Material, Figure S6B) or mitochondrial potential (Supplementary Material, Figure S6C).

**Figure 10:**
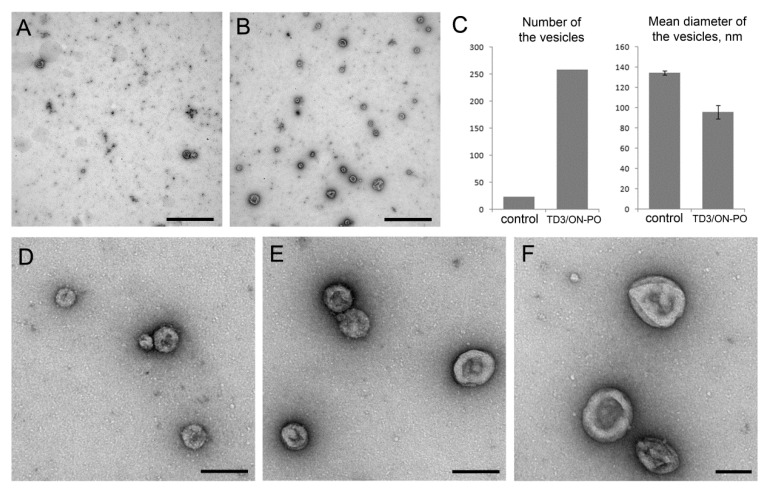
Transmission electron microscopic study of extracellular vesicles. (**A**,**B**) The abundance of vesicles in control cells and cells incubated with TD3/ON-PO duplexes, respectively. (**C**) The quantification of number and diameter of vesicles on three different (10 × 10 µm) areas of the grid. (**D**–**F**) Diameter variations of vesicles in the pellet from cells incubated with TD3/ON-PO duplexes. Scale bars: 1 µm (**A**,**B**), 100 nm (**D**–**F**).

Thus, it should be assumed that TD3/ON-PO duplexes observed in the extracellular space are included in some kind of vesicles produced by the transfected cells. To check this hypothesis, conditioned medium from KB-8-5 cells incubated in the presence of 1 µM of TD3/ON-PO duplexes for 16 h was sequentially centrifuged. The pellet collected at 16,000× *g* was analyzed by transmission electron microscopy (Figure 10). We found that, in contrast to the control cells, the pellet from cells incubated with TD3/ON-PO duplexes was enriched with vesicles of different sizes (Figure 10A,B).

Quantification of the vesicles revealed a high abundance of vesicles produced by cells incubated with TD3/ON-PO duplexes (Figure 10C). The diameter of these vesicles varied from 40–300 nm, with a mean value of 95.9 ± 1.9, whereas in control cells the diameter of vesicles varied from 80–200 nm, with a mean value of 134.4 ± 6.5 (Figure 10C–F). According to the morphological characteristics (cup-shaped) and the mean diameter (around 90 nm), we can assume that the majority of the vesicles secreted by TD3/ON-PO treated cells is represented by exosomes [62]. However, further investigations in this phenomenon are required.

### 2.8. Biological Activity of TD3/asON Duplexes

#### 2.8.1. Silencing of P-Glycoprotein Expression in KB-8-5 Cells by Duplexes TD3/ON-MDR1

In order to evaluate whether delivery using tONs provides a manifestation of the biological activity of the delivered oligonucleotide, we synthesized a series of antisense oligonucleotides targeting *MDR1* mRNA (Table 1; Supplementary Materials, Table S1). The sequence within *MDR1* mRNA to which the antisense oligonucleotides were directed was successfully used by us previously [56]. Drug resistant KB-8-5 cells that overexpress the *MDR1* gene and can grow in the presence of vinblastine were used to evaluate the silencing activity of duplexes tON/asONs since these cells are a convenient model to assess the biological activity of therapeutic nucleic acids [57,58,59,60].

At the first stage, we synthesized the asON-MDR1 antisense oligonucleotide and the complementary tON-MDR1 (Supplementary Materials, Table S1). However, preliminary experiments showed that asON-MDR1 delivered into the cells in the duplex with tON-MDR1 exhibited much lower silencing activity as compared with asON-MDR1 transfected by LF (data not shown). This low activity could be explained by the fact that the melting temperature of the duplex asON-MDR1/tON-MDR1 is 63.6 °C, and as such the stable duplex could not dissociate at 37 °C. These data correlate with the confocal microscopy investigation of cells loaded with TD3/dON duplexes of approximately the same stability that revealed almost complete co-localization of tON and dON, indicating absence of dissociation of the duplexes inside the cells.

To destabilize the duplex we introduced two or three mismatches in different sites of the tON, decreasing the T_m_ of its duplex with asON-MDR1 to 34–48 °C. However, the silencing activity of asON-MDR1 delivered by these mismatched duplexes, or delivered by LF but within asON/tON duplexes was still much lower than that of asON-MDR1 delivered by LF alone (data not shown). Thus, the destabilization of the duplex did not provide a sufficient concentration of free asON in the cell cytoplasm required for effective gene silencing. On the other hand, further decreases in T_m_ could have resulted in a dissociation of the tON from the duplex with the delivered oligonucleotide that was too fast, resulting in the failure of the asON accumulation in the cytoplasm of target cells.

Therefore, instead of asON-MDR1, we designed elongated *MDR1* mRNA targeting antisense oligonucleotides which consisted of (1) *MDR1* mRNA targeted asON, (2) a thymidine spacer containing four thymidine residues, and (3) a 17-mer oligonucleotide complementary to the TD3 transport oligonucleotide. The antisense part of the resulting oligonucleotide was either unmodified or was a gapmer flanked by three phosphorylguanidine modifications of the sugar-phosphate backbone at both the 5′- and 3′-ends (see Table 1, MDR1-ON and MDR1gap-ON, respectively).

**Figure 11:**
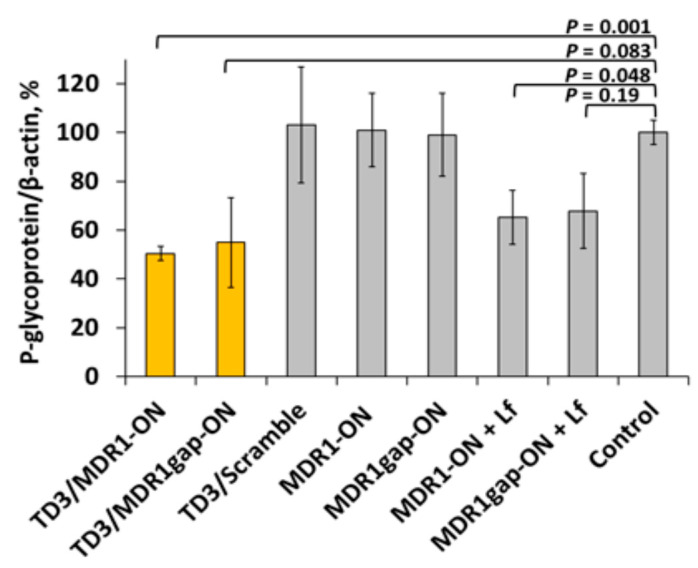
Silencing of P-glycoprotein expression in KB-8-5 cells by asONs delivered in the duplexes with TD3. The data were obtained using Western blotting carried out after 72 h of cell incubation. Human β-actin protein was used as an internal standard. Data were normalized to the ration of the P-glycoprotein/β-actin levels in untreated cells (control). Mean values (± SD) from three independent experiments are shown.

The biological activity of the duplexes of novel design, TD3/MDR1-ON and TD3/MDR1gap-ON, was studied in KB-8-5 cells grown in the presence of 300 nM vinblastine due to the overexpression of P-glycoprotein encoded by *MDR1* mRNA. The level of P-glycoprotein was estimated by Western blotting after 72 h incubation of cells with 6 µM TD3/MDR1-ON or TD3/MDR1gap-ON duplexes in the absence of the cytostatic in order to avoid the negative selection of living cells. The level of β-actin was used as an internal control. A scrambled oligonucleotide of the same design as MDR1-ON, but having no molecular targets in the human transcriptome, was used as control of specificity. The levels of P-glycoprotein were normalized to the levels of β-actin for data presentation (Figure 11). It was found that MDR1-ON delivered in cells by TD3 reduced the level of P-glycoprotein by 50% in comparison with scrambled oligonucleotide and non-treated control cells (Figure 11). The gapmer oligonucleotide, MDR1gap-ON, was delivered into cells in a similar way and exhibited a P-glycoprotein silencing activity similar to that of non-modified MDR1-ON oligonucleotide (Figure 11). Moreover, we showed that both MDR1-ON and MDR1gap-ON delivered by TD3 (Table 1) exhibited *MDR1* silencing activity close to the activity of the same oligonucleotides but delivered by Lipofectamine (Figure 11).

#### 2.8.2. Reversion of Multiple Drug Resistance Phenotype of KB-8-5 Cells by Antisense Oligonucleotides Delivered with TD3

**Figure 12:**
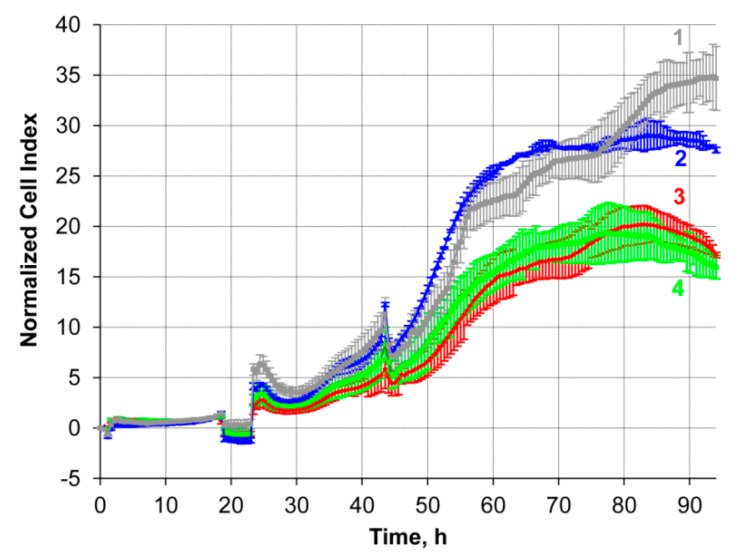
Restoration of sensitivity of KB-8-5 cells to vinblastine. The cell index of living KB-8-5 cells was estimated in real-time mode with an xCELLigence instrument. Cells were treated with 6 µM TD3/MDR1-ON or TD3/MDR1gap-ON duplexes for 24 h followed by addition incubation of cells in the presence of 300 nM vinblastine for 72 h. (1) control, intact cells; cells treated with (2) TD3/Scramble, (3) TD3/MDR1-ON, or (4) TD3/MDR1gap-ON duplexes (6 µM).

To confirm the efficiency of the transport oligonucleotide-based delivery system, the alterations in the sensitivity of drug-resistant KB-8-5 cells to vinblastine were estimated. KB-8-5 cells were incubated in the presence of duplexes TD3 with either *MDR1* targeted antisense oligonucleotide (MDR1-ON or MDR1gap-ON) or scrambled oligonucleotide (Scramble) (See Table 1) at a concentration of 6 µM. One day after addition of the duplexes, the cell medium was supplemented with 300 nM vinblastine and the sensitivity of cells to vinblastine was assessed in real-time with an xCELLigence instrument. The obtained data revealed that the growth rate of cells incubated with TD3/Scramble duplexes did not differ from the growth rate of intact cells (Figure 12, lines 1 and 2). Incubation of KB-8-5 cells with TD3/MDR1-ON and TD3/MDR1gap-ON duplexes resulted in a significant increase in the sensitivity of tumor cells to vinblastine and retarded tumor cell growth by approximately 40–45% in comparison with Scramble-treated cells (Figure 12, lines 3 and 4). Altogether, these results confirm both efficient intracellular delivery of asON by tONs and execution of silencing activity by the delivered antisense oligonucleotide. The level of cell sensitivity to vinblastine observed in these experiments was similar to that previously achieved by *MDR1* gene silencing with siRNA delivered by Lipofectamine 2000 or conjugates of siRNA with cholesterol penetrating into the cell in a carrier-free mode [58].

## 3. Discussion

In this study, a new approach has been developed that allows functional oligonucleotides to be delivered to cells with high efficiency while maintaining their biological activity. This approach is based on the formation of a duplex between an asON or other functional oligonucleotide containing modifications that ensure its stability with a tON carrying lipophilic or other residues to ensure penetration into the cell.

Two series of tONs were synthesized, in which dodecyl residues were attached to the 5′-end of the oligonucleotide strand through diamide-containing non-nucleotide inserts (TD-series) or anchored directly to the internucleotide phosphate (TP-series). To rationalize the scheme of tON synthesis we declined the post-synthetic modifications of tON with hydrophobic moieties and, instead, introduced dodecyl modifications during standard solid phase synthesis.

Simple hydrophobic dodecyl residues were chosen on purpose to tune the hydrophobicity of tONs by the variation in their number. Another benefit of dodecyl-containing derivatives is the significant increase in retention time upon HPLC isolation, which simplifies the isolation procedure. Yields of dodecyl-modified tON decreased from ~ 55% to ~ 40% and ~ 35% with increases in the number of dodecyl residues from one to three for both series of tONs (Supplementary Material, Figure S1). Further increases in the number of hydrophobic residues is likely to reduce the tON yield even more. It is unlikely that further increases in the number of hydrophobic tails above three could improve significantly the in vivo biodistribution of the duplexes, since such hydrophobic conjugates will interact mainly with LDL particles that guide the transport of oligonucleotides mainly to the liver [18,20]. It should be mentioned that using the scheme of synthesis utilized in our work, tONs can be modified with other hydrophobic molecules, for example, cholesterol.

The efficiency of penetration of developed tONs to cells depends directly on the number of dodecyl residues introduced into tONs regardless of the type of attachment of aliphatic molecules (TD or TP series), since no differences were observed in the intracellular accumulation of tONs bearing the same number of dodecyl residues but attached to either internucleotide phosphate or non-nucleotide inserts. tONs themselves, especially TD3 and TP3, efficiently accumulated in the cells with similar or even higher efficiency in comparison with oligonucleotides with Lipofectamine 2000-mediated delivery.

Duplexes of tONs/dONs were accumulated into the cells with somewhat lower efficiency in comparison with tONs alone, and this was apparently due to their higher molecular weight and total negative charge. Nevertheless, the level of intracellular accumulation of tONs/dONs duplexes was comparable with those observed in the presence of Lipofectamine 2000. It should be emphasized that dONs with different modifications of the sugar-phosphate backbone, such as phosphorothioate, phosphoryl guanidine modifications, or non-modified asONs, were delivered in duplexes with tONs with similar efficiencies. It was expected that dONs with phosphorothioate modifications could be delivered with a higher efficiency, but experiments demonstrated that the transfection efficiency was determined rather by the hydrophobic residues of tONs.

An important finding from our study is that transport oligonucleotides can deliver asONs into cells regardless of the type of the modification pattern of their sugar-phosphate backbone: unmodified, PS or PX oligonucleotides (Figure 5). The transfection activity of tONs (TD3 and TP3) was only slightly inferior in efficiency to Lipofectamine 2000-mediated ON-PO delivery (Figure 5). Thus, the proposed method of tON is working and provides in vitro transfection efficiency similar to the best protocols of lipofection.

The accumulation of duplexes measured by flow cytometry demonstrated different kinetics for tON and dON, in this case the ratio of the fluorescence intensity of cells at wavelengths corresponding to FAM and By5.5 gradually changed. However, confocal microscopy showed that tON and dON were co-localized during the entire observation. Since the duplexes used in the study of kinetics are stable at 37 °C, it can be expected that the emission of FAM and By5.5 fluorophores occurs in the same compartments. It has been shown that the fluorescence of FAM, in contrast to By5.5, is pH-sensitive and decreases significantly with decreasing pH [63], then the pH of the compartments with lipophilic conjugates can be indirectly estimated by the ratio of FAM/By5.5 fluorescence. We observed a slight decrease in the ratio during the kinetic study, and these ratios varied differently for duplexes with 2 and 3 hydrophobic tails. Based on the data on the decrease in FAM emission depending on pH and FAM/By5.5 ratios [63], it can be indirectly suggested that the localization of the duplexes with TD2 after 2 h is most likely in the compartments with pH 6 (early endosomes); after 4 h, in the compartments with 5.5 pH (late endosomes); and after 8 h, in the compartments with pH 4.5 (lysosomes). This estimated kinetics of trafficking correlates with the literature data reported previously for the fully modified PS asON delivered in a carrier-free mode [64,65]. Interestingly, in changing in the FAM/By5.5 ratios during the kinetic study, the duplexes with TD3 were higher than for those with TD2 and the main changes were observed 8 h after the addition of duplexes to the cells. Probably these differences are associated with the active process of recycling TD3 duplexes, since the recycling endosomes have a less acidic content, with a pH of 6.5; thus, the presence of duplexes in these compartments increase the average FAM/By5.5 ratio for the duplexes with TD3. We also observed a sharp decrease in the accumulation of duplexes with TD3 at the 8 h time point (Figure 7), which was accompanied by an increase in the number of extracellular vesicles detected by fluorescence microscopy (Figure 8) and transmission electron microscopy (Figure 10). Therefore, it can be concluded that duplexes with TD3, but not with TD2, are involved in active process of recycling.

Similar accumulation kinetics with a sharp decline as observed for TD3/dONs has been described in the literature for some other lipophilic nucleic acid conjugates. In particular, a 75% decrease in accumulation 5 h after addition to the cells has been observed for conjugates of siRNA and cholesterol in HeLa cells in the presence of serum [66]. A 25% reduction in accumulation 24 h post transfection after graduate augmentation was observed in the case of a conjugate of cholesterol and siRNA with a short single stranded tail containing PS modifications [67]. Such a decline is required for maintenance of cellular homeostasis, including constant concentrations of lipids and cholesterol, and is important for the survival and division of cells. Therefore, the maintenance of these parameters is ensured along with endocytosis by the reverse processes of recycling or exocytosis [68]. Thus, the decrease in the accumulation of lipophilic conjugates could be associated with a compensatory response of the cell to a high concentration of lipophilic molecules in the cytoplasm.

It should be noted that the delivery of asON as part of a duplex imposes certain limitations and puts forward additional requirements on the structure of the asON. The presence of a highly stable complex between asON and tON, which promotes efficient delivery, in turn, interferes with the implementation of the biological function, so we designed an elongated modular version of the antisense oligonucleotide. This duplex with a single stranded antisense part demonstrated similar silencing activity as anti-*MDR1* asONs transfected with LF. Moreover, TD3/MDR1-ON and TD3/MDR1gap-ON efficiently silenced the *MDR1* gene without LF (Figure 11) and the reverse phenotype of multidrug resistance of KB-8-5 cells (Figure 12). It should be noted that the use of such elongated modular asONs allows the use of the same tON to deliver a wide range of asONs directed to different sequences: in this elongated modular asONs the antisense part can be changed depending on the target sequence while tetra-thymidylate linker and tON complementary sequence will remain constant. An obvious advantage of the proposed system is that the sequence of the transport oligonucleotide does not require changes and remains constant for any RNA targets. In the future, it will be possible to design asONs containing non-nucleotide inserts or stimuli responsive bonds instead of the oligo-dT linker in the middle of the molecule, which will allow the antisense part to be cleaved from the duplex in certain compartments and, possibly, to avoid recycling.

It is known from literature data that an increase in the level of expression of *MDR1* by 1.5–2 times detected in clinical samples is already sufficient for the formation of a multiple drug resistance phenotype in tumor cells [69]. Therefore, a 50% reduction of p-glycoprotein levels by TD3/MDR1gap-ON is sufficient to restore the sensitivity of cells to vinblastine and to decrease the cell viability in the presence of vinblastine by 40–45% (Figure 3, Figure 4, and Figure 12). In vivo suppression of *MDR1* gene expression has been examined in a number of works [70,71,72,73,74] where xenograft models showed a decrease in tumor growth during chemotherapy combined with *MDR1* silencing. However, in these studies, the silencing of *MDR1* expression was achieved by using siRNA and the delivery was performed by liposomes, which may have side effects, including immunostimulation. The absence of toxicity of the developed delivery system in our experiments is promising to test the silencing and therapeutic potential of TD3/MDR1gap-ON in vivo in future experiments.

Thus, the developed tONs were proven to be efficient delivery vectors to transport asON to cells. Such an alternative delivery method allows for simplification and a reduction in the cost of asON delivery approach by reducing the complexity of conjugate synthesis and providing the possibility of using the entire arsenal of routine chemical synthesis. The proposed approach may allow the regulation of the hydrophobicity and other properties of tONs by the introduction of hydrophobic molecules and different functional groups, not only at the 5′-end, but also at the 3′-end or inside the oligonucleotides. Furthermore, the introduction of other hydrophobic and targeted molecules, such as PEG and cell-penetrating peptides, is possible through attachment to functional groups introduced into tON by the proposed method.

## 4. Materials and Methods

### 4.1. Oligonucleotide Preparation

Standard phoshoramidite solid-phase synthesis of all modified and unmodified oligonucleotides containing phosphodiester linkages (PO) was carried out on the ASM-800 DNA/RNA synthesizer (Biosset, Novosibirsk, Russia). Oligonucleotides were synthesized at 0.2 μmol scale, using standard commercial 2-cyanoethyl deoxynucleoside phosphoramidites and CPG solid supports (Glen Research, San Diego, CA, USA).

Oligonucleotides with internucleotide uncharged phosphoryl 1,3-dimethylimidazolidine-2-imino groups (PX) were synthetized as described by Kupryushkin et al. [27] and Stetsenko et al. [75].

Oligonucleotides containing phosphorothioate linkages (PS) were synthesized using sulfurizing reagent II (3-((dimethylaminomethylidene)amino)-3*H*-1,2,4-dithiazole-3-thione) (Glen Research, USA) 0.1 M in pyridine–acetonitrile 3:2 *v*/*v* according to the manufacturer’s protocol.

For introducing non-nucleosidic dodecyl-containing units into a TD-type of tON, the appropriate phosphoramidite, prepared as described in [48], was used as a 0.1 M solution in anhydrous acetonitrile, and the coupling time was extended from 1 to 10 min.

tON of TP-series containing phosphoramidate linkages (PN) at the internucleotidic phosphates were synthesized on a DNA/RNA synthesizer using a modified oxidation step. Oxidation was performed by addition of an iodine solution in pyridine (40 mM, 20 μL), followed by addition of 2.5 M dodecylamine in DMF/*N*,*O*-Bis(trimethylsilyl)acetamide (7:4, *v*/*v*, 20 μL). These two steps were repeated six times. The total time of the oxidation step was 12 min.

Labeling of oligonucleotides was performed using commercially available 5′-modifiers according to the manufacturer’s protocols. For labeling FAM and By5.5 2-Dimethoxytrityloxymethyl-6-(3′,6′-dipivaloylfluorescein-6-yl-carboxamido)-hexyl-1-*O*-[(2-cyanoethyl)-(*N*,*N*-diisopropyl)]-phosphoramidite (Glen Research, USA) or 2-[5-[3-butyl-1,1-dimethylbenzo[e]indol-2-ylidene]penta-1,3-dien-1-yl]-1,1-dimethyl-3-(5-{[4-{[(2-cyanoethoxy)(diisopropylamino)phosphanyl]oxy}cyclohexyl]carbamoyl}pentyl)benzo[e]indol-3-ium tetrafluoroborate (Cy5.5 analog, Promtech, Minsk, Belarus) were used, respectively. The sequences of the oligonucleotides are presented in Table 1.

### 4.2. Oligonucleotide Purification and Identification

Native and modified oligonucleotides were isolated by reverse-phased HPLC on an Agilent 1200 HPLC system (Santa Clara, CA, USA) using a Zorbax SB-C18 5 mm column 4.6 × 150 mm in a linear gradient of acetonitrile 0–50% or 0–90% in 20 mM triethylammonium acetate, pH 7.0, at flow rate of 2 mL/min. Fractions containing the desired product were pooled, concentrated in vacuo, dissolved in 0.1 mL of deionized water and precipitated by 1 mL of 2% LiClO_4_ in acetone. After centrifugation at 14,500 rpm for 2 min, washing with acetone and drying on air for 20 min, oligonucleotide pellets were dissolved in deionized water and stored at −20 °C. Oligonucleotide homogeneity was assessed by electrophoresis in PAAG 20%, 8 M urea, gel with 0.1 M Tris-borate, pH 8.3 as running buffer. The oligonucleotide bands were visualized in gel by staining with Stains-All (Sigma-Aldrich, Darmstadt, Germany).

Molecular masses of oligonucleotides were confirmed by LC-MS/MS ESI MS on an Agilent G6410A mass spectrometer (Santa Clara, CA, USA) in a negative ion mode. The samples were prepared by dissolving of oligonucleotides in 20 mM triethylammonium acetate in 60% aq. acetonitrile at a concentration of 0.1 mM. For phosphoryl guanidine oligonucleotides (PX), positive ion mode was applied. The PX samples (0.1 mM) were prepared by dissolving in 20 mM formic acid in 60% aq. acetonitrile. The volume of the samples was 10 µL. Analysis was carried out using 80% aq. acetonitrile as an eluent at a flow rate of 0.1 mL/min and using standard device settings. Molecular masses were calculated using experimental m/z values, obtained for each sample.

### 4.3. Duplex Thermal Denaturation Experiments

Thermal denaturation/renaturation experiments were carried out using a Cary 300 Bio spectrophotometer equipped with a Peltier temperature-controlled cuvette holder (Varian, Mulgrave, Victoria, Australia). Equimolar amounts (10 μM, 50 µL of each strand) of complementary oligonucleotides were used. Melting curves were recorded at 260 and 270 nm, within a temperature range from 5 °C to 95 °C with a heating/cooling rate of 0.5 °C/min, in a buffer consisting of 10 M sodium cacodylate pH 7.0 and 100 mM NaCl.

### 4.4. Cell Lines

KB-8-5 (human multidrug resistant cervical cancer cells), A549 (human lung carcinoma cells) and HEK293 (human embryonic kidney cells) were cultivated in DMEM medium supplemented with 10% FBS and 1% antibiotic/antimycotic mix (100 units/mL penicillin, 0.1 mg/mL streptomycin and 0.25 µg/mL amphotericin) at 37 °C in a humidified atmosphere with 5% CO_2_ (standard conditions, here and after SC) and passaged regularly to maintain exponential growth. In the case of KB-8-5, cell culture medium was additionally supplemented with 300 nM vinblastine.

### 4.5. Duplex Formation

Duplexes of tON with complementary dON were formed by heating equimolar amounts of oligonucleotides in PBS buffer at 95 °C for 3 min followed by incubation at room temperature until complete cooling. The obtained duplexes were used in experiments immediately after annealing.

### 4.6. Cell Viability Assay

Cytotoxicity of tON with respect to A549, HEK293 and KB-8-5 cells was monitored in real-time mode using the xCELLigence instrument (ACEA Biosciences, Santa Clara, CA, USA) for 48 h. Cells were seeded to adhere into 16-well E-plates (ACEA Biosciences, Santa Clara, CA, USA) at a density of 1.5 × 10^4^ cells/well in 150 µL/well DMEM medium supplemented with 10% FBS. E-plates with cells were placed into the xCELLigence instrument and incubated under SC for 24 h. On the next day, culture medium was replaced with 150 µL/well DMEM without serum and antibiotics and supplemented with 0.5, 1, 2.5, 5, 10, 25, and 50 µM of TD3. Cells were incubated in the xCELLigence instrument for 4 h under SC, then FBS was added to the cells up to a concentration of 10% and cells were incubated under SC for an additional 20 h. Cell index values were measured every 30 min. Dose-dependent curves were plotted for the time points 24 h post tON addition to the cells using MS Excel software. IC_50_ values were determined as the tON concentration required to decrease the cell index to 50% of the control cells (incubated in the absence of tON).

### 4.7. Effects of tON/asON Duplexes on Apoptosis Induction, Cell Cycle and Mitochondrial Potential

KB-8-5 cells were seeded to adhere into 24-well plates at a density of 1 × 10^5^ cells/well in 250 µL DMEM medium supplemented with 10% FBS in the absence of antibiotics and incubated for 20 h at SC. On the next day, culture medium was replaced with 200 µL serum- and antibiotic-free DMEM medium supplemented with 1 µM FAM-tON/By5.5-dON duplex, and cells were incubated for 4 h under SC. FBS was then added into the medium up to 10% and the cells were incubated for 12 h under SC. Apoptosis and cell cycle analysis was performed using an Annexin V-FITC Apoptosis Staining/Detection Kit (Abcam, Cambridge, UK) according to the manufacturer’s protocols. Briefly, the cells were detached from the plate with 2% trypsin (MP Biomedicals, Irvine, CA, USA), washed with PBS, resuspended in 200 µL of 1× binding buffer supplemented with 2 µL of Annexin V-FITC and 2 µL of propidium iodide (for apoptosis analysis) or 2 µL of propidium iodide alone (for cell cycle analysis). The cells were incubated for 5 min at room temperature in the dark, washed with PBS, fixed with 2% formaldehyde in PBS and analyzed on a NovoCyte flow cytometer (ACEA Biosciences, Santa Clara, CA, USA).

Mitochondrial membrane potential of KB-8-5 cells treated with tON/dON duplexes for 16 h was estimated using a MitoTracker Orange CMTMRos kit (ThermoFisher Scientific, Waltham, MA, USA) according to the manufacturer’s protocols. Briefly, after incubation with tON/dON duplexes, KB-8-5 cells were incubated in DMEM (serum- and antibiotic- free) medium supplemented with 0.2 µM MitoTracker for 30 min at 37 °C. After incubation, the cells were detached from the plate with 2% trypsin (MP Biomedicals, USA), centrifuged for 5 min at 300× *g*, washed with PBS, resuspended in PBS, and then analyzed on a NovoCyte flow cytometer (ACEA Biosciences, Santa Clara, CA, USA).

### 4.8. Analysis of Cellular Accumulation of tON or Duplexes of tON/dON

A549 or HEK293 cells were seeded into 24-well plates at a density of 10^5^ cells/well in DMEM medium supplemented with 10% FBS and left to adhere overnight. On the next day, culture medium was replaced with 250 µL/well DMEM without serum and antibiotics. FAM-tON or tON/FAM-dON duplexes formed as described above were added to the cells to a final concentration in each well of 1 µM and cells were incubated for 4 h under SC.

Upon analysis of the kinetics of intracellular accumulation of tON/dON duplexes, KB-8-5 cells were incubated in DMEM with 1 µM FAM-tON/By5.5-dON duplexes in the absence of serum and antibiotics for 4 h under SC; then FBS was added to the cells to a concentration of 10% and cells were incubated for 20 h under SC.

### 4.9. Flow Cytometry

Flow cytometry was used to characterize the levels of intracellular accumulation of the oligonucleotides labeled with fluorophores (transfection efficiency, TE), namely either FAM-tON or the duplexes of FAM-tON/By5.5-dON. The TE of tON or duplexes was estimated after 4 h of incubation (for A549 and HEK293 cells) or after 1, 2, 4, 8, 16, and 24 h of incubation (for KB-8-5 cells). After incubation, cells were detached from the plate with 2% trypsin (MP Biomedicals, Irvine, CA, USA), resuspended in complete DMEM, centrifuged at 200 g for 5 min, washed with PBS and fixed with 2% formaldehyde in PBS buffer (10 min, room temperature). Cells were analyzed on a NovoCyte flow cytometer (ACEA Biosciences, Santa Clara, CA, USA) and data was processed with NovoExpress (ACEA Biosciences, Santa Clara, CA, USA). All experiments were run in triplicate for statistical analysis. The TE was characterized by two values: percentage of fluorescent-positive cells in a population and mean fluorescent intensity (MFI) of cells in a sample.

### 4.10. Confocal Microscopy

KB-8-5 cells were seeded on coverslips and placed into 24-well plates at a density of 6 × 10^4^ cells/well in DMEM medium containing 10% FBS, 300 nM vinblastine, and 1% antibiotics and incubated overnight under SC. Then, cell medium was replaced by DMEM without FBS and antibiotics and FAM-tON or duplexes FAM-tON/By5.5-dON were added to each well (1 µM). Cells were incubated with tON or duplexes for 1, 2, and 4 h in the absence of FBS in the medium. At the 4 h time point, FBS was added to cells up to a concentration of 10% and cells were further incubated for 4, 12, and 20 h under SC. After incubation, coverslips with cells were washed with PBS and placed on slides in a drop of DAPI/Antifade (Millipore). Intracellular localization of tON or duplexes was assessed with confocal fluorescent microscopy on LSM710 (Zeiss, Oberkochen, Germany), using a plan-apochromat 63×/1.40 Oil DIC M27 objective. Analysis of intracellular accumulation of tON/dON duplexes and Z-stack were performed using ZEN software (Zeiss, Oberkochen, Germany). Confocal analysis was performed in three channels (blue, green, red). Fluorescence in the blue channel corresponded to DAPI (nuclei staining); the green channel corresponded to fluorescence of tON labeled with FAM (FAM-TD3 or FAM-TD2) and the red channel corresponded to dON labeled with By5.5 (By5.5-ON-PO or By5.5-ON-PX).

### 4.11. Transmission Electron Microscopy of Extracellular Vesicles Containing Duplexes

KB-8-5 cells were seeded to adhere onto 60 mm Petri dishes at a density of 5 × 10^5^ cells in 3 mL of DMEM medium supplemented with 10% FBS in the absence of antibiotics and incubated for 20 h at SC. On the next day, culture medium was replaced with 3 mL of serum- and antibiotic-free DMEM supplemented with 1 µM FAM-tON/By5.5-dON duplexes and cells were incubated for 4 h under SC. Then FBS was added into the medium up to a concentration of 10% and the cells were incubated additionally for 12 h under SC. Next, the conditioned medium was collected and sequentially centrifuged at (1) 200× *g*, 10 min, 4 °C (to remove detached cells); (2) 2000× *g*, 20 min, 4 °C (to remove cell debris); (3) 16,000× *g*, 1 h, 4 °C (to precipitate microvesicles). Then, the supernatant was discarded, the microvesicle pellet was resuspended in PBS buffer and centrifuged for 30 min, at 16,000× *g* and 4 °C. Finally, the pellet was stored at 4 °C and processed for negative staining with a 1% aqueous solution of uranyl acetate and electron microscopy. A 5 µL drop of buffer with gently resuspended microvesicles was applied to the glow-discharged copper grid and drawn off with filter paper. Then, the grid was placed onto a drop of uranyl acetate solution for a few seconds and the excess liquid was removed with filter paper. The last step was repeated keeping the grid on the drop for 30 s. The grids were left to dry and then analyzed with a transmission electron microscope (Jeol JEM 100-SX). The diameter of the vesicles was measured on three different areas of the grid (10 × 10 µm) with ImageJ software (v.1.5) [76].

### 4.12. Western Blotting

The level of P-glycoprotein in KB-8-5 cells was evaluated in a vinblastine-free medium. The cells in the exponential phase of growth were plated in 48-well plates (10^4^ cells/well) and incubated for 24 h. Then, growth medium was replaced by fresh DMEM without FBS supplemented with 6 µM TD3/Scramble, TD3/MDR1-ON or TD3/MDR1gap-ON duplexes and cells were incubated for 4 h under SC. Next, FBS was added into the medium up to a concentration of 10% and the cells were incubated additionally for 72 h under SC. The cells were lysed in 60 µL sample buffer (Sigma-Aldrich, Darmstadt, Germany). A total of 10 µL of each sample was loaded onto a 10% SDS/polyacrylamide gel and then separated at 60 mA for 1 h. The proteins were transferred from PAAG to a PVDF membrane (Millipore, Burlington, NJ, USA) using a tank criterion blotter (Bio-Rad, USA) and the membrane was then blocked overnight in 2% non-fat dried milk in PBS. The membranes were incubated with monoclonal anti-P-glycoprotein and anti-β-actin antibodies (Sigma-Aldrich, Darmstadt, Germany) at 1:1000 and 1:3000 dilutions, respectively, for 1 h. Afterwards, the membranes were washed in PBS with 0.1% Tween-20 and subsequently incubated for 1 h with secondary rabbit anti-mouse antibodies conjugated with horseradish peroxidase (Invitrogen, Waltham, MA, USA). Luminescence detection was performed by Versadoc 4000 MP (Bio-Rad, Hercules, CA, USA) using a chemiluminescent reagent kit (Abcam, Cambridge, UK). Human β-actin protein was used as an internal control. Data were analyzed using GelPro 4.0. software (Media Cybernetics, Rockville, MD, USA).

### 4.13. Evaluation of Sensitivity of KB-8-5 Cells to Vinblastine

Sensitivity of KB-8-5 cells loaded with tON/asON duplexes to vinblastine was estimated by monitoring cell survival for 120 h in real time mode with an xCELLigence instrument (ACEA Biosciences, Santa Clara, CA, USA). KB-8-5 cells were seeded in 16-well E-plates at a density of 5 × 10^4^ cells per well and incubated overnight. Then, culture medium was replaced with fresh DMEM without FBS and antibiotics, and supplemented with 6 µM tON/asON duplexes, and cells were incubated for 4 h under SC. Next, FBS and antibiotics were added to the concentrations of 10% and 1%, respectively. Cells were cultured for 24 h under SC followed by 300 nM vinblastine addition and incubation for 96 h under SC.

### 4.14. Statistical Analysis

The data were statistically processed using one-way ANOVA. Post-hoc testing was completed using Fisher’s least significant differences (LSD). *p* < 0.05 was considered to be significant. The statistical package STATISTICA version 10.0 (TIBCO Software, Palo Alto, CA, USA) was used for analysis.

## Figures and Tables

**Table 1:** Sequences, designations, and modifications of transport and antisense oligonucleotides used in the study.

Type of Oligonucleotide	Name	Sequence and Modifications ^1^	M_r_ (Theor)/M_r_ (Exp)
Transport Oligonucleotides	TD1 FAM-TD1	5′-T[R_1_]-GGTAGCAAGTCGAGACT-3′ 5′-[FAM]p[R_1_]-GGTAGCAAGTCGAGACT-3′	n.d. ^2^ 6233.47/6232.40
	TD2 FAM-TD2	5′-T[R_1_][R_1_]-GGTAGCAAGTCGAGACT-3′ 5′-[FAM]p[R_1_][R_1_]-GGTAGCAAGTCGAGACT-3′	n.d. 6639.93/6639.00
	TD3 FAM-TD3	5′-T[R_1_][R_1_][R_1_]-GGTAGCAAGTCGAGACT-3′ 5′-[FAM]p[R_1_][R_1_][R_1_]-GGTAGCAAGTCGAGACT-3′	6783.10/6782.00 7046.39/7044.50
	TP1 FAM-TP1	5′-[R_2_]-GGTAGCAAGTCGAGACT-3′ 5′-[FAM]p[R_2_]-GGTAGCAAGTCGAGACT-3′	5731.05/5723.16 6298.54/6296.50
	TP2 FAM-TP2	5′-[R_2_][R_2_]-GGTAGCAAGTCGAGACT-3′ 5′-[FAM]p[R_2_][R_2_]-GGTAGCAAGTCGAGACT-3′	6202.59/6201.14 6770.08/6768.12
	TP3 FAM-TP3	5′-[R_2_][R_2_][R_2_]-GGTAGCAAGTCGAGACT-3′ 5′-[FAM]p[R_2_][R_2_][R_2_]-GGTAGCAAGTCGAGACT-3′	6674.13/6673.00 7241.62/7241.00
Delivered Oligonucleotides	ON-PO FAM-ON-PO By5.5-ON-PO	5′-AGTCTCGACTTGCTACC-3′ 5′-[FAM]pAGTCTCGACTTGCTACC-3′ 5′-[By5.5]pAGTCTCGACTTGCTACC-3′	5121.41/5119.43 5688.90/5688.12 5906.42/5905.60
	ON-PS FAM-ON-PS	5′-A^S^G^S^T^S^C^S^T^S^C^S^G^S^A^S^C^S^T^S^T^S^G^S^C^S^T^S^A^S^C^S^C-3′ 5′-[FAM]pA^S^G^S^T^S^C^S^T^S^C^S^G^S^A^S^C^S^T^S^T^S^G^S^C^S^T^S^A^S^C^S^C-3′	5378.44/5377.12 5945.94/5944.31
	ON-PX FAM-ON-PX By5.5-ON-PX	5′-A^X^G^X^T^X^C^X^T^X^C^X^G^X^A^X^C^X^T^X^T^X^G^X^C^X^T^X^A^X^C^X^C-3′ 5′-[FAM]pA^X^G^X^T^X^C^X^T^X^C^X^G^X^A^X^C^X^T^X^T^X^G^X^C^X^T^X^A^X^C^X^C-3′ 5′-[By5.5]pA^X^G^X^T^X^C^X^T^X^C^X^G^X^A^X^C^X^T^X^T^X^G^X^C^X^T^X^A^X^C^X^C-3′	6643.78/6641.90 7211.28/7208.50 8037.22/8035.00
Antisense Oligonucleotides	MDR1-ON	5′-GTCCAGCCCCATGGA-TTTT-AGTCTCGACTTGCTACC-3′	
	MDR1gap-ON	5′-G^X^U^X^C^X^CAGCCCCAU^X^G^X^G^X^A-TTTT-AGTCTCGACTTGCTACC-3′	11721.24/11721.60
	Scramble	5′-G^X^U^X^U^X^CCTCGCGCU^X^C^X^C^X^A-TTTT-AGTCTCGACTTGCTACC-3′	11651.13/11647.80

^1^ Structures of groups: **S**—phosphorothioate group; **X**—phosphoryl guanidine group. ^2^ n.d.—not determined. 
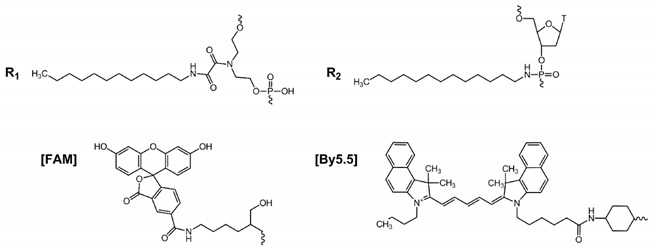

## Supplementary Materials

The following are available online. Figure S1: HPLC profiles of transport oligonucleotides; Figure S2: Cytotoxicity of TD3 transport oligonucleotide; Figure S3: Accumulation of duplexes TD3/ON-PX in KB-8-5 cells; Figure S4: Accumulation of duplexes TD2/ON-PX in KB-8-5 cells; Figure S5: Extracellular localization of FAM-TD3/By5.5-ON-PO duplexes; Figure S6: Influence of TD3/ON-PO duplexes on apoptosis, cell cycle and mitochondrial potential of KB-8-5 cells; Table S1: Additional list of oligonucleotides used in preliminary experiments on *MDR1* gene silencing.
