# Supplementary material for: Transport Oligonucleotides—A Novel System for Intracellular Delivery of Antisense Therapeutics
Source: Molecules. 2020 Aug 11;25(16):3663. doi: 10.3390/molecules25163663 (PMC7464317; doi:10.3390/molecules25163663)
Supplement: Supplementary file 1 [file molecules-25-03663-s001.pdf]

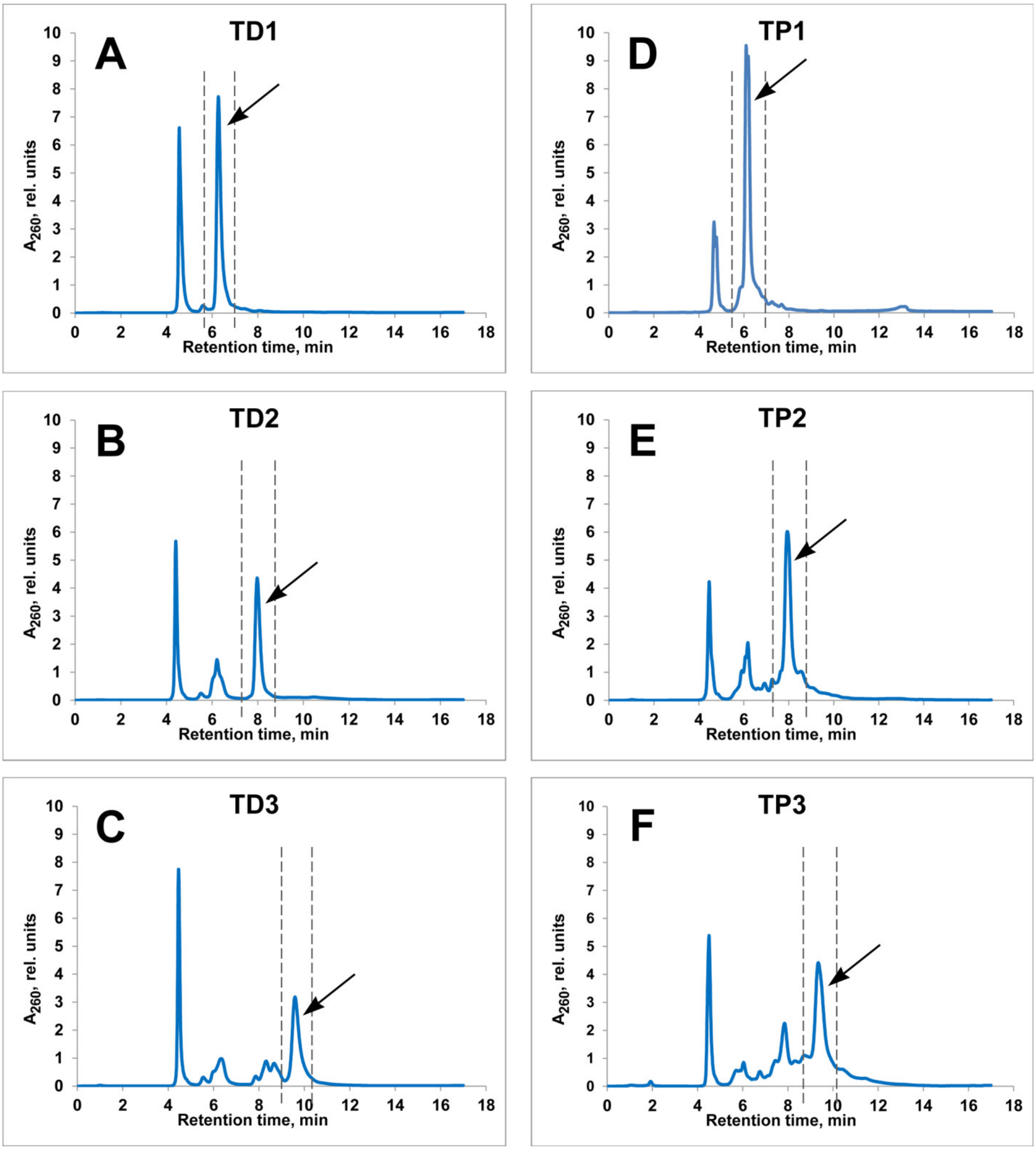


**Figure S1.** HPLC profiles of transport oligonucleotides. A – TD1. B – TD2. C – TD3. D – TP1. E – TR2. F – TP3.

The hydrophobicity of tONs depends significantly on the number of dodecyl residues introduced into oligonucleotide. This is why in each case the product with the highest retention time (the most hydrophobic one) is the desired one.

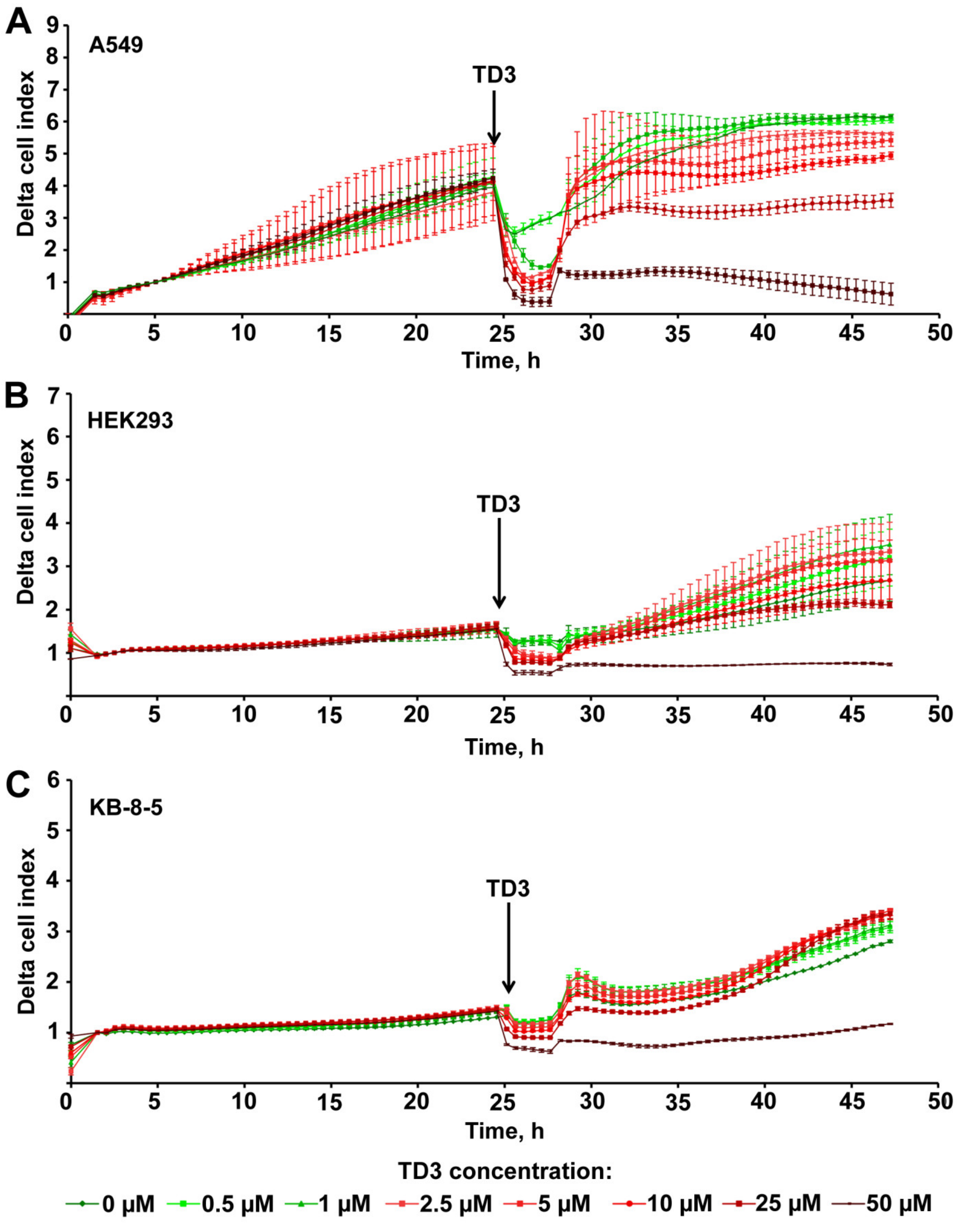


**Figure S2.** Cytotoxicity of TD3 transport oligonucleotide with respect to (A) A549, (B) HEK293 and (C) KB-8-5 cells monitored in real-time with an xCELLigence instrument. Cells were seeded to adhere overnight followed by a change of culture medium supplemented with TD3 oligonucleotide at concentrations from 0.5 to 50 μM and the additional incubation of cells for 4 h. Then FBS was added to the cells, up to concentration of 10%, and cells were incubated for 20 h. Data are presented as MEAN ± SD. Each experimental point represents experiments run in triplicate.

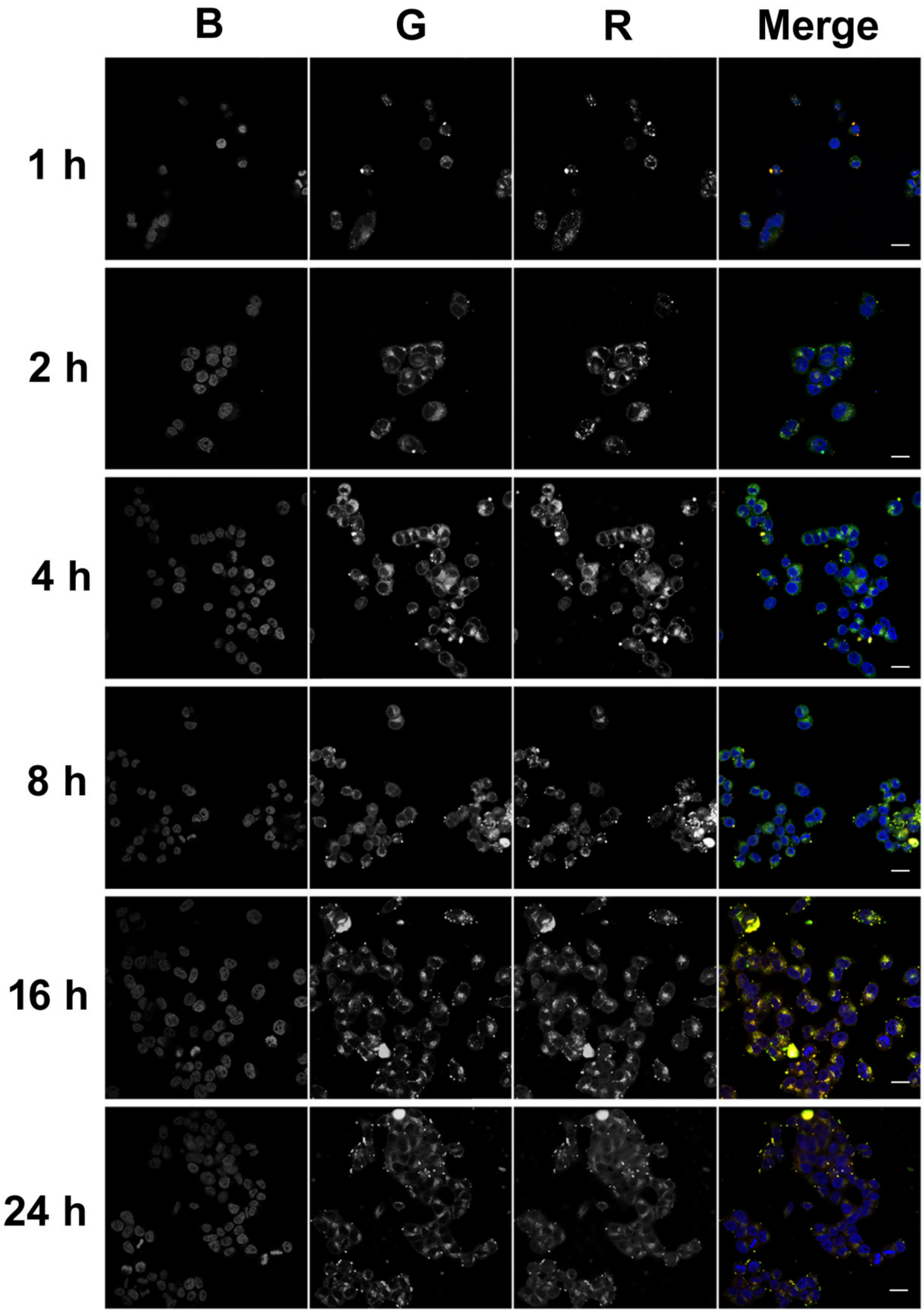


**Figure S3.** Accumulation of duplexes TD3/ON-PX in KB-8-5 cells. Analysis of samples was performed 1, 2, 4, 8, 16 and 24 h after addition of duplexes (1 μM) to the cells. Duplexes consisted of FAM-labeled TD3 and By5.5-labeled ON-PO. Analysis was performed with confocal fluorescent microscopy by using a Plan-Apochromat 63×/1.40 Oil DIC M27 objective. Three-channel (BGR) pictures were obtained using staining by DAPI (nuclei staining) (B); FAM (G), attached to TD3 and By5.5 (R), attached to ON-PO. Scale bars: 20μm.

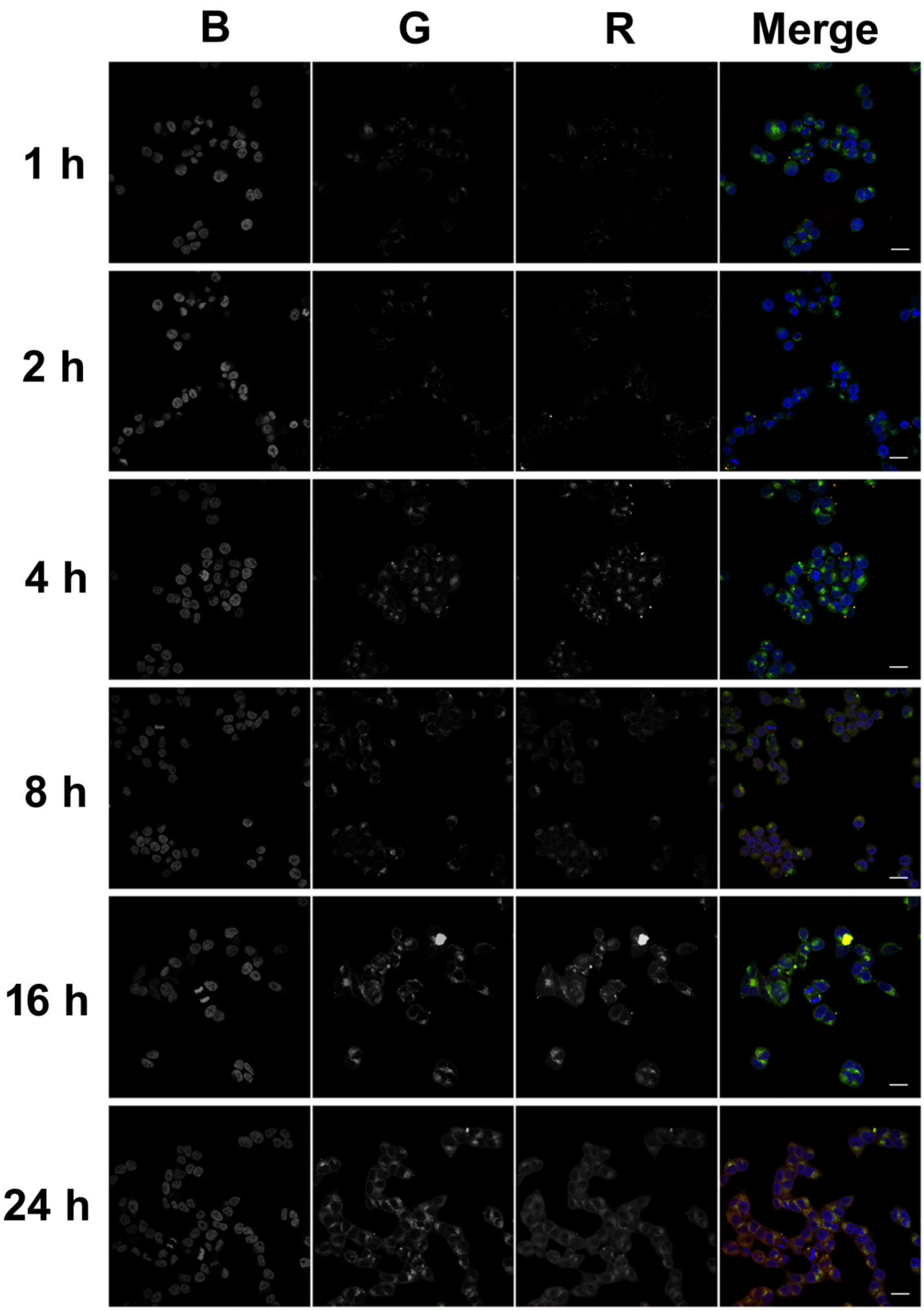


**Figure S4.** Accumulation of duplexes TD2/ON-PX in KB-8-5 cells. Analysis of samples was performed 1, 2, 4, 8, 16 and 24 h after addition of duplexes (1 μM) to the cells. Duplexes consisted of FAM-labeled TD2 and By5.5-labeled ON-PX. Analysis was performed with confocal fluorescent microscopy by using a Plan-Apochromat 63×/1.40 Oil DIC M27 objective. Three-channel (BGR) pictures were obtained using staining by DAPI (nuclei staining) (B); FAM (G), attached to TD2 and By5.5 (R), attached to ON-PX. Scale bars: 20μm.

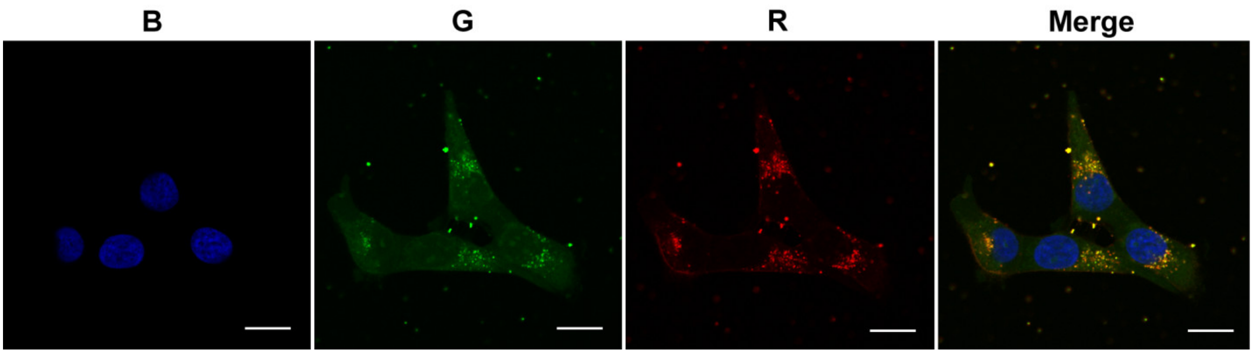


**Figure S5.** Extracellular localization of FAM-TD3/By5.5-ON-PO duplexes after incubation of KB-8-5 cells with duplexes for 8 h. Cells were incubated with duplexes for 4 h in medium without FBS, then thoroughly washed with PBS and additionally incubated for 4 h in medium supplemented with 10% FBS. Analysis was performed with confocal fluorescent microscopy (100× magnification). Three-channel (BGR) pictures were obtained using staining by DAPI (nuclei staining); (B), FAM (G), attached to TD3 and By5.5 (R), attached to ON-PO.

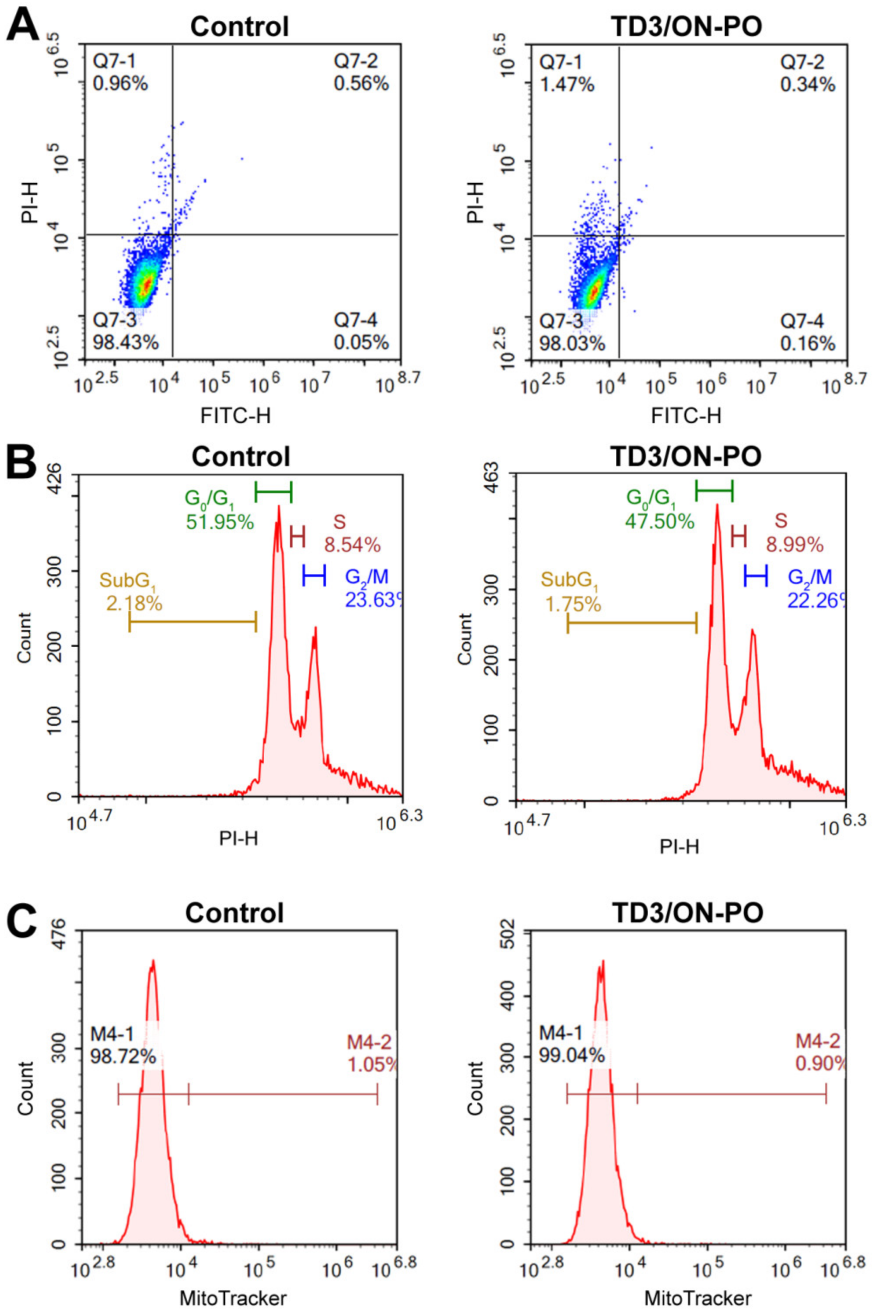


**Figure S6.** Influence of TD3/ON-PO duplexes on (A) apoptosis, (B) cell cycle and (C) mitochondrial potential of KB-8-5 cells incubated with duplexes for 16 h.

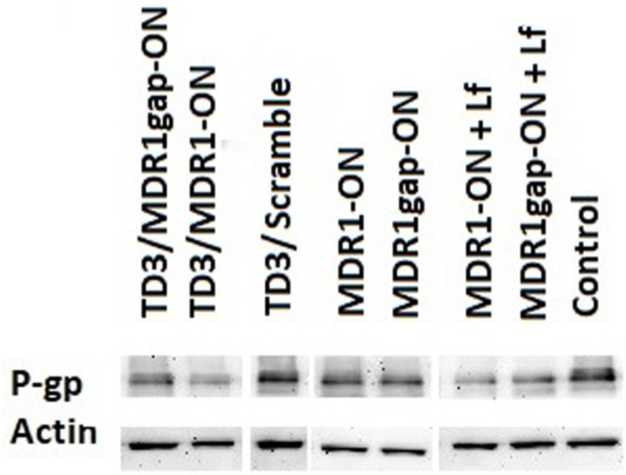


**Figure S7.** Silencing of P-glycoprotein expression in KB-8-5 cells by asONs delivered in the duplexes with TD3. Representative western blot showing *p*-glycoprotein expression levels after 72 h of cell incubation with the duplexes. Human β-actin protein was used as an internal standard.

**Table S1.** Additional list of oligonucleotides used in preliminary experiments on *MDR1* gene silencing.

| Designation | Sequence 5′ – 3′ | Tm of duplex with asON-MDR1 | |
|---|---|---|---|
| | | Calculated | Measured |
| asON-MDR1 | GTCCAGCCCCATGGA | | |
| tON-MDR1 | TCCATGGGGCTGGAC | 64.4 | 63.6 ± 0.1 |
| tON-MDR1(m1) | TC**T**ATGGGG**T**TGGAC | 43.1 | 48.4 ± 0.4 |
| tON-MDR1(m2) | T**TT**ATGGGG**T**TGGAC | 34.1 | 47.2 ± 0.4 |
| tON-MDR1(m3) | TCCAT**T**G**T**GCTGGAC | 33.8 | 35.4 ± 0.8 |
| tON-MDR1(m4) | TCCAT**TTTT**CTGGAC | - | 33.8 ± 7.9 |

Mismatches are presented in red, **bold** and underlined
